# Detection of rare nematode resistance *Rhg1* haplotypes in *Glycine soja* and a novel *Rhg1* α‐SNAP

**DOI:** 10.1002/tpg2.20152

**Published:** 2021-10-30

**Authors:** Derrick J. Grunwald, Ryan W. Zapotocny, Seda Ozer, Brian W. Diers, Andrew F. Bent

**Affiliations:** ^1^ Dep. of Plant Pathology Univ. of Wisconsin–Madison Madison WI 53706 USA; ^2^ Dep. of Crop Science Univ. of Illinois at Urbana–Champaign Urbana IL 61801 USA

## Abstract

This study pursued the hypothesis that wild plant germplasm accessions carrying alleles of interest can be identified using available single nucleotide polymorphism (SNP) genotypes for particular alleles of other (unlinked) genes that contribute to the trait of interest. The soybean cyst nematode (SCN, *Heterodera glycines* [HG]) resistance locus *Rhg1* is widely used in farmed soybean [*Glycine max* (L.) Merr.]. The two known resistance‐conferring haplotypes, *rhg1‐a* and *rhg1‐b*, typically contain three or seven to 10 tandemly duplicated *Rhg1* segments, respectively. Each *Rhg1* repeat carries four genes, including *Glyma.18G022500*, which encodes unusual isoforms of the vesicle‐trafficking chaperone α‐SNAP. Using SoySNP50K data for *NSF_RAN07_* allele presence, we discovered a new *Rhg1* haplotype, *rhg1‐ds*, in six accessions of wild soybean, *Glycine soja* Siebold & Zucc. (0.5% of the ∼1,100 *G. soja* accessions in the USDA collection). The α‐SNAP encoded by *rhg1‐ds* is unique at an important site of amino acid variation and shares with the *rhg1‐a* and *rhg1‐b* α‐SNAP proteins the traits of cytotoxicity and altered N‐ethylmaleimide sensitive factor (NSF) protein interaction. Copy number assays indicate three repeats of *rhg1‐ds*. *G. soja* PI 507613 and PI 507623 exhibit resistance to HG type 2.5.7 SCN populations, in part because of contributions from other loci. In a segregating F_2_ population, *rhg1‐b* and *rhg1‐ds* made statistically indistinguishable contributions to resistance to a partially virulent HG type 2.5.7 SCN population. Hence, the unusual multigene copy number variation *Rhg1* haplotype was present but rare in ancestral *G. soja* and was present in accessions that offer multiple traits for SCN resistance breeding. The accessions were initially identified for study based on an unlinked SNP.

## Core Ideas

- Germplasm carrying useful alleles can be identified using SNP data for other genes.
- *Glycine soja* accessions with potentially valuable SCN resistance were identified.
- A novel variant of the *Rhg1* α‐SNAP SCN resistance protein was identified and characterized.
- The unusual *Rhg1* multigene copy number variation structure arose prior to the domestication of soybean.

**Abbreviations**
FI: female index
HC: high‐copy
HG: *Heterodera glycines*
LC: low‐copy
NSF: N‐ethylmaleimide sensitive factor
ORF: open reading frame
PCR: polymerase chain reaction
QTL: quantitative trait loci
RAC: *rhg1‐a* α‐SNAP copia
SCN: soybean cyst nematode
SNARE: soluble NSF attachment protein receptor
SNP: single nucleotide polymorphism.

## 1 INTRODUCTION

Productive enhancements in feed and food crops are often driven by phenotypic selection during conventional plant breeding, but phenotype alone does not always reveal the donor material with the best potential for trait diversification and improvement (Tuberosa, 2012). Recent developments, such as single nucleotide polymorphism (SNP) genotyping and next generation sequencing have made it possible to prescreen germplasm for desirable traits through correlation with sequence information (Poland et al., 2012). In some cases, this approach can be extended to wild relatives of crop plants. Novel alleles of loci of agronomic importance may then be subjected to functional characterization. Alleles of interest can be moved into elite varieties via sexual crosses or transgenic approaches.

Soybean [Glycine max (L.) Merr.] is one of the world's most important legume crops, providing a major source of vegetable oil, protein meal for animal feed, and potential sources of renewable energy (http://soystats.com/) (Graham & Vance, 2003; Stacey, 2010. Soybean cyst nematode (SCN) (*Heterodera glycines* [HG]) is the most economically damaging pathogen of soybean, routinely causing upwards of US$1 billion each year in the United States alone (Davis et al., 2008; Koenning & Wrather, 2010; Mitchum, 2016). Soybean cyst nematode eggs can remain viable for years in cysts, which are recalcitrant to many environmental or chemical conditions, making control of established SCN populations difficult (Niblack et al., 2006). Resistant varieties and crop rotation are the major methods of SCN control. *Glycine soja* is the wild ancestor of cultivated soybean, and the more diverse gene pool in *G. soja* species vs. domestic soybean offers a source of novel alleles or genes for traits of agronomic interest including SCN resistance (Hyten et al., 2006; Liu et al., 2020.

The strong‐effect quantitative trait locus (QTL) *Rhg1* (*Resistance to Heterodera glycines 1*) is the most used source of SCN resistance, with the *rhg1‐b* haplotype from PI 88788 present in the vast majority of SCN‐resistant soybean grown in the United States. (Colgrove & Niblack, 2008; Concibido et al., 2004; Donald et al., 2006. Intriguingly, *Rhg1* is a tandemly repeated block of four genes, and the number of repeats varies from one in susceptible soybean varieties or three in most *rhg1‐a* haplotypes to nine or 10 copies in *rhg1‐b* haplotypes (Cook et al., 2012). None of the genes in the repeated block are reminiscent of canonical defense or disease resistance genes. Both silencing and overexpression experiments have established a role in SCN resistance for three of the four genes in the *Rhg1* locus, one of which (*Glyma.18G022500*) encodes an α‐SNAP (alpha‐soluble N‐ethylmaleimide sensitive factor (NSF) attachment protein) (Cook et al., 2012).

The α‐SNAP is a functionally conserved eukaryotic protein that interacts in multimeric complexes with both NSF and soluble NSF attachment protein receptor (SNARE) proteins to mediate vesicular trafficking (Jahn & Scheller, 2006). In particular, α‐SNAP and NSF cooperate to promote vesicle trafficking through their disassembly for recycling of the bundled v‐ and t‐SNARE complexes that form during vesicle fusion (Jahn & Scheller, 2006). In soybean, we recently discovered that *Rhg1‐*encoded α‐SNAPs are unusual in that they bind poorly to wild‐type NSF (Bayless et al., 2016). In *Nicotiana benthamiana* Domin transient assays, expression of *rhg1‐a* or *rhg1‐b* α‐SNAPs disrupts vesicle trafficking and is cytotoxic, eventually causing cell death (Bayless et al., 2016, 2018). Furthermore, these aberrant α‐SNAPs accumulate approximately tenfold in syncytial cells as a response to SCN, suggesting a role of vesicle trafficking efficiency in the outcome of SCN–soybean interactions (Bayless et al., 2016, 2019). Soybean haplotypes containing three *Rhg1* repeats (low‐copy [LC] haplotypes; *rhg1‐a*; Peking‐type or Hartwig‐type) encode a distinct α‐SNAP protein while those haplotypes containing nine to 10 *Rhg1* repeats (high‐copy [HC] haplotypes; *rhg1‐b*; PI 88788‐type) encode a second distinct α‐SNAP protein (Cook et al., 2014). To maximize SCN resistance, varieties carrying LC *rhg1‐a* haplotypes require presence of additional QTL at chromosome 11 (associated with a loss‐of‐function intron retention allele at a chromosome 11 α‐SNAP gene) and at chromosome 8 (*Rhg4*, encoding a serine hydroxymethyltransferase) (Bayless et al., 2018; Lakhssassi et al., 2017; Liu et al., 2012. These and other studies (Lakhssassi et al., 2020; Liu et al., 2017; Patil et al., 2019; Yu et al., 2016) have identified additional attributes shared by or distinct between the *rhg1‐a* vs. *rhg1‐b* haplotypes.

**Core Ideas**

- Germplasm carrying useful alleles can be identified using SNP data for other genes.
- *Glycine soja* accessions with potentially valuable SCN resistance were identified.
- A novel variant of the *Rhg1* α‐SNAP SCN resistance protein was identified and characterized.
- The unusual *Rhg1* multigene copy number variation structure arose prior to the domestication of soybean.

Linkage disequilibrium has been observed between the soybean chromosome 18 *Rhg1* locus and a chromosome 7 locus, with segregation distortion observed only in genotypes carrying an SCN resistance‐associated *Rhg1* allele (Webb et al., 1995; Kopisch‐Obuch & Diers, 2005; Vuong et al., 2015). Our group recently found that this distortion is attributable to a unique NSF allele that is encoded at *Glyma.07G195900* on chromosome 7 (Bayless et al., 2018). Termed *NSF_RAN07_* (for *Rhg1*‐associated NSF on chromosome 7), this allele is present in 11% of 19,645 soybean accessions in the USDA collection but remarkably is present in all soybean accessions and segregating progeny that are homozygous for the SCN resistance‐associated *rhg1‐a* or *rhg1‐b* haplotypes. At the apparent α‐SNAP–NSF binding interface, the encoded NSF_RAN07_ protein carries atypical amino acids at sites that become proximal to the unusual amino acids that distinguish α‐SNAP_Rhg1_LC and α‐SNAP_Rhg1_HC. The NSF_RAN07_ protein exhibits higher affinity than wild‐type NSF for binding those resistance‐associated α‐SNAP proteins and enables the viability of soybeans carrying *Rhg1*‐encoded SCN resistance (Bayless et al., 2018).

The gradual evolution of SCN populations toward HG types (or races) that partially or largely overcome the SCN resistance in commercially grown soybean varieties (McCarville et al., 2017; Niblack et al., 2008) has motivated searches for new SCN resistance sources. Specific *G. soja* accessions have already provided new sources of SCN resistance that show promise (Brzostowski et al., 2017; Usovsky et al., 2020; Yu & Diers, 2017). In a recent study by Lee et al. (2015b), whole‐genome sequencing revealed but did not further investigate a tandem duplication of *Rhg1* in *G*. *soja* accession Jidong 5 (W06), suggesting that resistance‐conferring *Rhg1* haplotypes may have arisen prior to the divergence of *G. max* and *G. soja*. Further research on *Rhg1* duplications in *G. soja* accessions can provide insights into the evolution of SCN resistance.

In the present study, we used a SNP marking the physically unlinked but genetically associated *NSF_RAN07_* allele to discover and examine diversity of *Rhg1* in *G. soja* germplasm available through the USDA collection. We identified infrequent *G. soja* accessions containing tandem repeat copies of the *Rhg1* locus that represent an apparent progenitor of soybean *rhg1‐a* and *rhg1‐b*. We found that the α‐SNAP encoded at *G. soja* multicopy *Rhg1* loci is unique, yet carried structural and functional similarities to the *rhg1‐a* and *rhg1‐b* α‐SNAPs. Partially as a result of contributions from other loci, specific *G. soja* accessions carrying *NSF_RAN07_* and *rhg1‐ds* were found to also carry strong resistance to problematic HG type 2.5.7 SCN.

## 2 MATERIALS AND METHODS

### 2.1 Plant materials

Seeds of 22 *G. soja* or *G. max* (the first 22 entries in Supplemental Table S1) were obtained from the USDA Soybean Germplasm Collection in Urbana, IL (https://www.ars‐grin.gov/). LD10‐10198 is an elite *G. max* experimental line developed at the University of Illinois that carries the *rhg1‐b* haplotype for SCN resistance from PI 88788

### 2.2 DNA extractions and oligonucleotide primers

Soybean genomic DNA was extracted from young cotyledons from respective *G. soja* or *G. max* accessions using standard CTAB extractions as in Keim et al. (1988) with modifications from Cook et al. (2012). The oligonucleotide primers used for polymerase chain reaction (PCR) and other assays are listed in Supplemental Table S3.

### 2.3 Copy number variation assays

**FIGURE 1:**
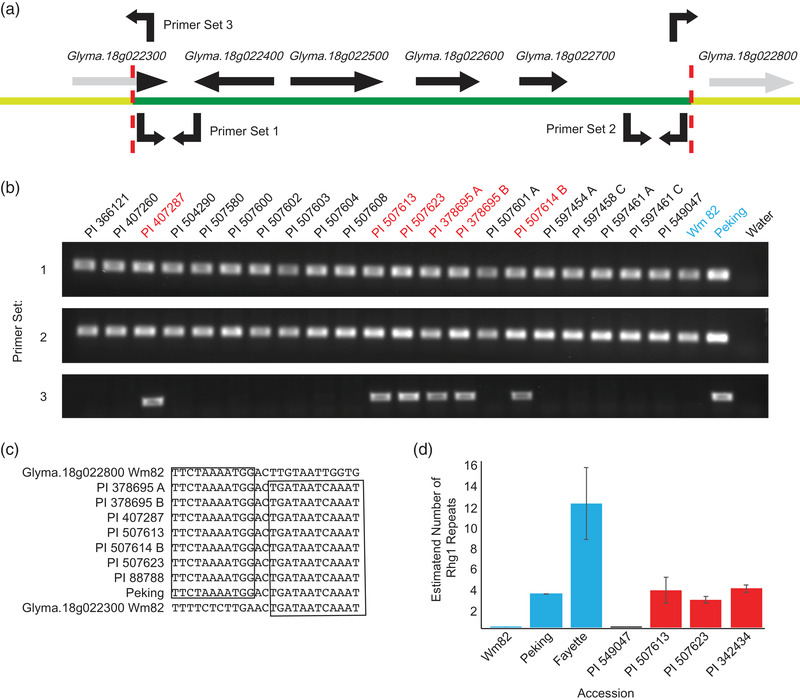
Multiple *Glycine soja* carry the *Resistance to Heterodera glycines 1* (*Rhg1*) repeat junction. (a) Schematic of polymerase chain reaction (PCR) primers used in (b). (b) Results of PCR using Primer Set 3 primers that orient outwards from within the 5′ and 3′ ends of the ∼31 kb segment of *Rhg1* found in both single‐ and multicopy lines. Product indicates tandem duplication of the *Rhg1* locus. (c) DNA sequence of the *Rhg1* repeat junction found in *Glycine soja* accessions that contain a *Rhg1* duplication event. (d) *Rhg1* copy number qPCR on *G. soja* accessions as well as domesticated soybean cultivars Wm82, Peking, and Fayette. Error bars show standard error of the mean

Copy number variation assays were performed essentially as in Lee et al., 2015b. In brief, 80 ng of extracted genomic DNA was subjected to qPCR using primers directed against the bridge junction as in Figure 1 or *Glyma.18G022800* using SolisBiodyne 5× Firepol (SolisBiodyne; Cat. No. 08‐36‐00001). Copy number was estimated using the ΔCt method where ΔCt is the difference between *Glyma.18G022800* and bridge amplifications. For TaqMan assays, experiments were performed essentially as in Lee et al. (2016).

### 2.4 Targeted DNA extraction

DNA was enriched for the *Rhg1* locus using 837 specific patch oligos as in Varley et al. (2010) with significant modifications. DNA was first digested using three combinations of restriction enzymes: DraI/TaqαI/NlaIII, Mn1I/MboII, and ApoI/HpyCH4V (100 ng DNA per reaction). A patch‐driven ligation was performed with 2 nM of each complementary oligo (Supplemental Table S3), 40 nM nested forward, 20 nM ‘U2’, 20 nM ‘U2‐Phospho’, 5 U of ampligase (Epicentre Cat. No. A3202K), and 1× ampligase buffer in 25 μl. This reaction was incubated for 10 min at 95 °C, followed by 30 sec at 95 °C and 4 min at 54 °C for 80 cycles. Undesired products and excess genomic DNA was then digested with the direct addition of 5 U exonuclease I (USB) and 100 U exonuclease III (Epicentre Cat. No. EX4425K). This was incubated at 37 °C for 1 h followed by inactivation at 95 °C. Products were purified using 42 μl (1.6×) of Agencourt AMPure XP beads (Beckman Coulter Cat. No. A63880) and eluted in 20 μl ddH20. A nested PCR was then performed using KAPA HiFi HotStart Uracil+ Readymix (2×) (KapaBiosystems Cat. No. KK2801), 100 μM of the oligos ‘right‐no homology’ and ‘left‐no homology’, 2.5 mM MgCl_2_ and 20 μl of the eluted product from previous step. This reaction was incubated according to manufacturer instructions with an annealing temperature of 66 °C and 30 s extension for 31 cycles. Product was again purified using 1.6× sample volume of Agencourt AMPure XP beads before submission for sequencing at the University of Wisconsin Biotechnology Center facility. Samples were individually barcoded before being sequenced using Illumina MiSEquation (Illumina) 2 × 250 with 50% PhiX.

### 2.5 Sequence variant detection

Flow cell data were demultiplexed and Illumina adapter sequences trimmed by the University of Wisconsin Biotechnology Center facility. Universal sequences were trimmed using Cutadapt (v2.8; Martin, 2011). Previously published whole‐genome sequencing read data from soybean HG type indicator lines as well as the *G. soja* genotypes W05 were also added to the analysis moving forward. (Cook et al., 2014, Bioproject PRJNA243933 [HG type test lines]; Xie et al., 2019, Bioproject PRJNA486704[W05]). Full genome alignment was performed as in Cook et al. (2014) using the Wm82.a2.v1 reference genome (Schmutz et al., 2010) using the programs BWA (v0.7.17, Li & Durbin, 2010) and Picard (v2.19). The HaplotypeCaller function with the GATK software package (Poplin et al., 2017) was used to detect variants in the genomic region Gm18:1632666‐1663881. Because of the nature of our targeted DNA extraction, we lacked complete coverage of the locus. Because of this, we identified regions within *Rhg1* where all the sequenced *G. soja* samples had depth in excess of three. Vcftools (v1.13, Danecek et al., 2011) was used to pull out variants within these regions and filter for a depth of three and variant quality value of 50. A fasta sequence file was obtained for each sample using the FastaAlternateReferenceMaker function within GATK. These fasta files were then imported to SplitsTree for network analysis (Huson & Bryant, 2006).

### 2.6 Phylogenetic analysis and phylogenetic trees

To determine the relationship between *G. soja* and *G. max* or among *G. soja*, we aligned all the SNPs available in the SoySNP50K database (Song et al., 2013, 2015). The program SplitsTree (v4.16.1) was used to perform the alignment and construct the network (Huson & Bryant, 2006). Uninformative sites were excluded in the network construction. Rooted phylogenetic trees were built using MEGAX (Kumar et al., 2018; Stretcher et al., 2020). The rooted phylogeny for the *Rhg1* locus was performed using DNA sequences described in the preceding section, corresponding to bp Gm18:1,636,000‐1,659,000 of *G. max* genome assembly version Glyma.Wm82.a2.

### 2.7 RNA isolation and cDNA synthesis

Total RNA was extracted from expanding soybean trifoliates using either the DirectZol RNA miniprep plus kit (Zymo Research) or the RNeasy mini kit (Qiagen) using manufacturer's instructions. RNA samples were DNase treated, and total RNA was quantified using spectrophotometry. Integrity of RNA was checked by visualization of rRNA bands on a 1.2% agarose gel. cDNA synthesis was performed using the iScript cDNA synthesis kit (Bio‐Rad) with ∼1 μg RNA as input. The Peking soybean accession used in these studies was PI 548402.

### 2.8 Vector construction

The *G. soja* α‐SNAP alleles (*Glyma.18G022500*) from each of the six multicopy *Rhg1 G. soja*, and the NSF (*Glyma.07G195900*), SHMT*_Rhg4_* (*Rhg4*; *Glyma.08G11490*), α‐SNAP_Ch11_ (*Glyma.11G234500)*, and 5′ NSF open reading frame (ORF) from *G. soja* accession PI 507623, were amplified from generated cDNA (for α‐SNAP_Rhg1_, NSF and *Rhg4*), or genomic DNA (for α‐SNAP_Ch11_ and 5′ NSF), using Kapa HiFi polymerase (Roche). They were placed directly under the control of the soybean ubiquitin promoter and nopaline synthase terminator in pBlueScript using Gibson assembly (Gibson et al., 2009) and subsequently sequence verified. For the α‐SNAP_Rhg1_ and the NSF, the promoter‐ORF‐terminator expression cassette was digested with XbaI and PstI or NotI and SalI (New England Biosciences), respectively, and the fragments were purified using the Zymoclean large fragment DNA recovery kit (Zymo Research). Purified DNA fragments were then ligated into the pSM101 binary vector (Melito et al., 2010) using T4 DNA ligase (New England Biosciences).

For sequencing of the bridge junction, the NSF_Ch07_ amplicon and the last exon of α‐SNAP_Ch11_,the *G. soja Rhg1* bridge junction, *NSF_Ch07_*, or α‐SNAP_Ch11_ amplicon was amplified from genomic DNA using GoTaq green (Promega) and cloned into pGEM‐T Easy using ligation per manufacturers recommendations (Promega). Constructs were verified by colony PCR and diagnostic digest before being sequenced by Sanger sequencing.

### 2.9 Transient *Agrobacterium* expression in *Nicotiana benthamiana*

*Agrobacterium tumefaciens* strain GV3101 (pMP90) containing the indicated expression cassettes was infiltrated using a blunt tip tuberculin syringe at an OD_600_ = 0.8 into young leaves of *N. benthamiana* plants. The GV3101 cultures were grown overnight at ∼28 °C in media containing 25 μg ml^−1^ kanamycin and 100 μg ml^−1^ rifampicin and induced for ∼3 h in 10 mM MES (pH = 5.6), 10 mM MgCl_2_ and 100 mM acetosyringone prior to leaf infiltration. *N. benthamiana* plants were grown at 25 °C with a photoperiod of 16 h light at 100 μE m^−2^ s^−1^ and 8 h dark.

### 2.10 Antibodies and immunoblotting

Generation and validation of rabbit polyclonal antibodies raised against *Rhg1* α‐SNAP_LC_, *Rhg1* α‐SNAP_HC_, and α‐SNAP_WT_ was previously described in Bayless et al. (2016). Tissue preparation and immunoblots were performed essentially as in Bayless et al. (2016, 2018). Bradford assays were performed on each leaf or root extract supernatant and equal amount of OD_595_ total protein were loaded onto SDS–PAGE gels. Immunoblots for α‐SNAP and NSF were incubated overnight at 4 °C in 5% nonfat dry milk TBS‐T (50 mM Tris, 150 mM NaCl, 0.05% Tween 20) at 1:1000. Secondary horseradish peroxidase conjugated goat antirabbit IgG (Sigma) was added at 1:10,000 and incubated for 1 h at room temperature on a platform shaker followed by five washes with TBS‐T. Chemiluminescence signal detection was performed with SuperSignal West Dura chemiluminescent substrate (Thermo Scientific) and developed using a Chemi Doc MP chemiluminescent imager (Bio‐Rad).

For antibody sensitivity assays, purified recombinant α‐SNAP was serially fivefold diluted to concentrations of 800, 160, and 32 pg. The proteins were then loaded onto an SDS–PAGE gel and immunoblots performed as above. To confirm loading of gel lanes, the gel was stained using ProteoSilver Kit (Sigma) according to manufacturer's instruction.

### 2.11 Recombinant proteins

A vector encoding the ORF of the *G. soja* ortholog of *Glyma.18G02250* from PI 507623 was generated, and recombinant protein expression and purification were performed as in Bayless et al. (2016). In brief, the ORF was cloned into the pRham‐N‐His‐SUMO expression vector, protein was purified using PerfectPro Ni‐NTA resin as per manufacturer's instruction, His and SUMO tags were cleaved by incubating the dialyzed protein with 1 unit per 100 μg protein of SUMO express protease (Lucigen) and removed by rebinding to the Ni‐NTA column, and the recombinant protein was eluted in SNAP freeze‐down buffer (50 mM Tris, 50 mM NaCl, 10% (w/v) glycerol, 0.5 mM TCEP, pH = 8). Both purity and quantity of protein were evaluated by Coomassie blue staining relative to BSA standards.

### 2.12 In vitro binding assays

In vitro binding assays were performed as previously described (Bayless et al., 2016). In brief, 20 μg of the specified recombinant α‐SNAP was added to the bottom of a 1.5 mL polypropylene tube, unbound α‐SNAP was removed with wash buffer, and 20 μg of either NSF or NSF_RAN07_ in NSF binding buffer (20 mM HEPES, 2 mM EDTA, 100 mM KCl, 500 μM ATP, 1 mM DTT, 1% (w/v) PEG 4000) was added and incubated on ice for 10 min. The solution was then removed and excess NSF was removed by washing twice with NSF binding buffer. Samples were subsequently boiled in 1× SDS loading buffer and separated on a 10% Bis‐Tris SDS–PAGE gel. To detect bound NSF, the gel was stained using the ProteoSilver Kit (Sigma) according to manufacturer's instruction and quantified relative to α‐SNAP abundance by densitometric analysis using ImageJ.

### 2.13 Nematode resistance tests

The nematode resistance tests were performed according to Niblack et al. (2009) with modifications described by Yu et al. (2016). In brief, the genotypes LD10‐10198 and PI 507623 were crossed in a greenhouse. The F_1_ seeds were generated and grown to maturity to produce an F_2_ population. Ninety‐six F_2_ plants from the cross were evaluated in a resistance bioassay.

In the first SCN bioassay, seed of the SCN indicator lines (Niblack et al., 2009), the *G. soja* accession, and the susceptible check ‘Lee 74′ were germinated on germination paper. After 3 d, individual seedlings were transplanted into PVC tubes filled with steam‐sterilized sandy soil and packed in plastic crocks. Each tube was infested with ∼2,000 SCN eggs derived from either HG type 0 or HG type 2.5.7 nematode populations. The experimental unit was a tube with an individual plant and the tubes were randomized using a completely randomized design. The test with each isolate was done separately and there were three replicates of the indicator lines and six replicates of the PI. After 30 d, cysts were collected by washing roots over a 250 μm sieve. The cysts were then counted, and the female index (FI) was determined using the following equation: FI (%) = (number of female cysts on an entry) / (average number of female cysts on susceptible control ‘Lee 74′) × 100. The F_2_ population was evaluated for resistance to a HG type 2.5.7 population according to the procedures listed above. In this test, each F_2_ plant was placed in a separate tube and 10 plants of the susceptible control Lee 74 was included. DNA was isolated from a leaf sample taken from each F_2_ plant according to Yu et al. (2016) and tested with the genetic marker Satt309 according to Kim et al. (2010). A single factor analysis of variance was completed to test for an association between the genetic marker and FI.

## 3 RESULTS

### 3.1 A small number of *Glycine soja* accessions contain a multicopy *Rhg1* locus

As an entry point to identify *G. soja* accessions that potentially carry resistance alleles at *Rhg1*, we searched for accessions that carry genetic markers associated with the *G. max NSF_RAN07_* allele at *Glyma.07G195900*. *NSF_RAN07_* is essential for the viability of soybean plants carrying the cyst nematode resistance *Rhg1* haplotypes *rhg1‐a* and *rhg1‐b* (Bayless et al., 2018). The SoySNP50K Infinium BeadChip had previously been used to generate genetic data for over 19,000 domesticated soybean and over 1,000 wild soybean accessions from 84 countries, testing over 47,000 SNP loci in each accession (Song et al., 2013, 2015). In the absence of strongly predictive *Rhg1* markers in the SoySNP50K dataset, we used SNP ss715597431 that is specific for the R_4_Q polymorphism in the encoded NSF_RAN07_ protein that was recently shown to be necessary for the viability of *G. max* that carry multicopy *Rhg1* haplotypes (Bayless et al., 2018). When we queried the SoySNP50K dataset for accessions annotated as *G. soja* that contain this SNP, only 21 lines had the corresponding polymorphism, representing ∼1.7% of the ∼1,100 *G. soja* annotated accessions (Supplemental Table S1). We hypothesized that some of these accessions might also contain *Rhg1* haplotypes that confer SCN resistance.

The *Rhg1* locus was then examined in the 21 selected *G. soja* accessions. Prior work has indicated that *Rhg1*‐mediated resistance in *G. max* is driven substantially by the presence of three to 10 tandem duplicate copies of the >30 kb *Rhg1* locus, in so‐called LC (*rhg1‐a*) and HC (*rhg1‐b*) genotypes (Cook et al., 2014, 2012; Lee et al., 2015b). Lee et al. (2015b) noted a *G. soja* accession (W06/Jidong5) with three copies of the *Rhg1* locus. The vast majority of soybean accessions in the USDA collection, including the cultivar Williams 82, are SCN‐susceptible and carry the single‐copy *Rhg1* haplotype (Bayless et al., 2019; Cook et al., 2012; Lee et al., 2015b). To test the hypothesis that the R_4_Q‐containing *G. soja* also carry multicopy *Rhg1* loci, we developed *Rhg1* repeat junction PCR primers as in Cook et al. (2012) but specific for *G. soja*. Primer pairs were first designed (Figure 1a) to amplify the 5′ and 3′ terminal portions of *Rhg1*, to confirm amplification of *Rhg1*, and allow DNA sequencing of the *G. soja* products. A PCR assay was then used to test for the *Rhg1* repeat junction by pairing one outward‐directed PCR primer for each terminus of the *Rhg1* repeat, which should only give a product if the 3′ terminus of one *Rhg1* repeat segment is adjacent to the 5′ terminus of the next *Rhg1* repeat. Screening of the 21 *G. soja* accessions revealed that six contain a duplicated *Rhg1* (Figure 1b). Use of a different oligonucleotide pair, and a limited set of changes to the template preparation and PCR conditions, produced the same results regarding presence of the repeat junction PCR product in these accessions (Supplemental Figure S1a).

### 3.2 The *Glycine soja* multicopy *Rhg1* locus shares structural characteristics with *Glycine max* multicopy *Rhg1* haplotypes

The *Rhg1* repeat junction was previously shown to be conserved between HC and LC SCN‐resistant *G. max* genotypes, suggesting a shared lineage (Cook et al., 2014, 2012; Lee et al., 2015b). To test whether these multicopy *Rhg1 G. soja* loci also share that lineage, we cloned and sequenced the repeat junction from the six repeat‐positive genotypes shown in Figure 1b. Those *G. soja* accessions were found to carry a *Rhg1* repeat junction that is identical to that of the *rhg1‐a* soybean genotype Peking and the *rhg1‐b* soybean accession PI 88788 (Figure 1c). As previously reported, the *Rhg1* repeat junction is not present in the single‐copy *Rhg1* of the *G. max* reference genome for Williams 82 (Figure 1c). The above observations support the hypothesis that all known multicopy *Rhg1* types in annual *Glycine* species arose from a shared event. Because of the differences from *G. max* multicopy *Rhg1* haplotypes we describe later, and to follow upon established use of the allele terms *rhg1‐a*, *rhg1‐b*, and *rhg1‐c* for previously described soybean haplotypes, we named this *G. soja* multicopy *Rhg1* haplotype ‘*rhg1‐ds’*, short for *rhg1‐d (soja)*.

**FIGURE 2:**
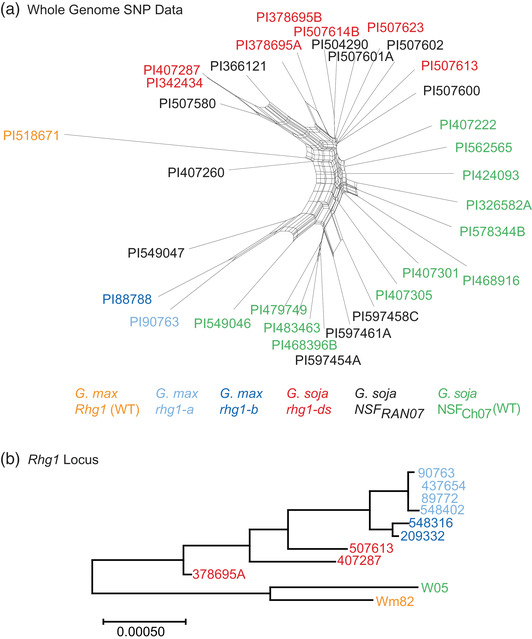
Multicopy *Resistance to Heterodera glycines 1* (*Rhg1*)‐containing *Glycine soja* group separately from multicopy *Rhg1*‐containing *G. max* at the levels of whole genome and *Rhg1* locus. (a) Unrooted phylogeny assessing relatedness of whole‐genome single nucleotide polymorphism (SNP) signatures of a representative set of *G. soja* accessions. Accessions containing *rhg1‐ds* and *NSF_RAN07_* (red) were analyzed alongside those containing *NSF_RAN07_* but lacking the multicopy *rhg1‐ds* (black) and a representative semirandom set of non‐*rhg1‐ds* non‐*NSF_RAN07_ G. soja* accessions (green). Note that *G. max* accessions carrying *rhg1‐a* or *rhg1‐b* also carry *NSF_RAN07_*. (b) Rooted phylogeny generated using genomic sequences across 23 kb of the *Rhg1* locus from the denoted accessions comparing relatedness of the *rhg1‐a* (light blue), *rhg1‐b* (dark blue), and *rhg1‐ds* (red) loci as well as the *Rhg1* loci from accession W05 (a *G. soja* carrying a single‐copy *Rhg1*; green) and *G. max* cv. Williams 82 (orange)

As it is possible that *G. soja* accessions carrying the *rhg1‐ds* haplotype arose through hybridization events, we performed unrooted phylogenetic analyses (Huson & Bryant, 2006). Phylogenetic analyses were first conducted using genome‐wide SNP data from the SoySNP50K dataset (Song et al., 2013, 2015), to assess the overall relatedness of accessions carrying NSF_RAN07_ and *rhg1‐ds*, to each other and to a more broadly representative set of *G. soja* accessions. The comparison set was a relatively random set of *G. soja* accessions chosen because they have been included in recent publications from other research groups (Figure 2a; Supplemental Figure S2a). At the whole‐genome scale, *G. soja* accessions carrying an NSF_RAN07_–encoding allele are dispersed throughout the phylogeny (Figure 2a). The rare *rhg1‐ds*‐containing *G. soja* accessions were more narrowly clustered, suggesting a shared derivation within *G. soja*. Unsurprisingly, individual *rhg1‐ds*‐containing *G. soja* also can be closely related to *G. soja* that do not carry *rhg1‐ds* (Figure 2a). At a genome‐wide level the *rhg1‐ds‐*containing *G. soja* group separates from *G. max* that carry *rhg1‐a* or *rhg1‐b*, suggesting that *rhg1‐ds* arose independent of these haplotypes rather than from a recent hybridization with *G. max* that carry *rhg1‐a* or *rhg1‐b* (Supplemental Figures S2a and S2b).

To study the relatedness of the *rhg1‐ds* locus to the *rhg1‐a* and *rhg1‐b* loci, we used a targeted approach to extract and sequence genomic DNA from *Rhg1* resulting in >70% coverage of the *rhg1‐ds* loci from PI 507613, PI 407287, and PI 378695A. These accessions were chosen because they are relatively distant from each other in the phylogenetic analysis (Figure 2a). The data were compared with previously determined *Rhg1* sequences from *G. max* and *G. soja*. Figure 2b shows that *rhg1‐ds* forms a clade with *rhg1‐a* and *rhg1‐b* that is distinct from the more common wild‐type (single‐copy, SCN‐susceptible) haplotypes. The *Rhg1* loci from the susceptible *G. max* and *G. soja* are more similar to each other than to *rhg1‐a*, *rhg1‐b*, or *rhg1‐ds*. Although the shared origin of soybean *rhg1‐a* and *rhg1‐b* and *G. soja rhg1‐ds* was already implied from their identical *Rhg1* repeat junction sequences (Figure 1c), the phylogeny shown in Figure 2b further demonstrates their apparent shared derivation. The data also suggest that *rhg1‐ds* arose prior to the split between *rhg1‐a* and *rhg1‐b*.

We selected two *rhg1‐ds G. soja* accessions, PI 507613 and PI 507623 (Figure 2a), to characterize in further detail. They were collected in 1983 as wild plants, ∼80 km from each other, in central Japan. We also continued to study *rhg1‐ds*‐containing PI 342434 because, although presently annotated in the USDA germplasm collection as a *G. max*, it carries leaf shape and plant architecture traits that are intermediate between *G. max* and *G. soja* and contains a genome‐wide SNP pattern that clusters with *G. soja* accessions (Figure 2a). PI 342434 is also from Japan (donated to USDA in 1969) and was originally annotated as a *Glycine ussuriensis* (Regel & Maack) ‘tsurumame’ edible wild soybean. We sought to determine the *Rhg1* copy number of these three accessions using both a genomic DNA qPCR method and a copy number variation TaqMan assay as in Lee et al. (2016, 2015b). Our implementation of the TaqMan assay (example shown in Supplemental Figure S1b) was unsuccessful, as we obtained erratic data even for controls, but those assays did indicate a *rhg1‐ds* copy much lower than in *rhg1‐b* cultivar Fayette and similar to that of *rhg1‐a* cultivar Forrest. Genomic qPCR assays (Figure 1d) were more reproducible and also included domesticated soybean cultivars of known *Rhg1* copy number 1, 3, and 10 as controls. The tested *G. soja* and *G. max* had approximately the same number of copies of *Rhg1* as Peking (three copies; Figure 1d). This is consistent with the hypothesis that after multicopy *Rhg1* haplotypes became established, higher soybean *Rhg1* copy number soybeans such as *rhg1‐b* with nine or 10 copies may have been a trait derived under positive selection during breeding (Lee et al., 2015b).

### 3.3 *Glycine soja* multicopy *Rhg1* encodes a distinct α‐SNAP

**FIGURE 3:**
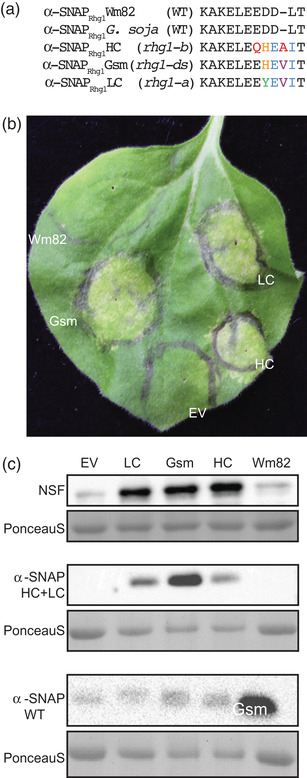
Unique *Glycine soja* alpha‐Soluble NSF Attachment Protein (α‐SNAP) induces cell death and N‐ethylmaleimide Sensitive Factor (NSF) hyperaccumulation in *Nicotiana benthamiana*. (a) Alignment of C‐terminal amino acids of the *Resistance to Heterodera glycines 1* (*Rhg1*) α‐SNAP from multicopy *rhg1‐ds Glycine soja* (α‐SNAP_Rhg1_Gsm) compared with *Rhg1* α‐SNAPs from single‐copy SCN‐susceptible cv. Williams 82 (Wm82; wild type [WT]) or *G. soja* W05 (*G. soja*; WT), and multicopy SCN‐resistant *G. max* cultivars. PI 88788 (high copy [HC]; *rhg1‐b*) and Peking (low copy [LC]; *rhg1‐a*). (b) Representative *N. benthamiana* leaf 7 d after infiltration with *A. tumefaciens* expressing empty vector negative control or the denoted *Rhg1* α‐SNAP with no epitope tag [abbreviations as in (a)]. (c) Immunoblots detecting levels of endogenous *N. benthamiana* NSF or α‐SNAP (α‐SNAP‐WT), as well as recombinant *Glycine* α‐SNAPs delivered via *A. tumefaciens* as in (b). Same sample was used for entire columns in (c) but probed with three separate antibodies. Leaf tissue was harvested ∼60 h after infiltration; Ponceau S staining indicates relative levels of total protein in each sample

Investigation of the *G. soja rhg1‐ds* locus revealed that it encodes a novel *Rhg1* α‐SNAP variant (Figure 3a). The contributions of *Rhg1* α‐SNAP proteins to SCN resistance depend heavily on the presence of altered sets of C‐terminal amino acid residues at sites that are otherwise highly conserved across multicellular eukaryotes (Bayless et al., 2016; Cook et al., 2014). Distinct sets of C‐terminal amino acids are carried by each of the *Glyma.18G022500 Rhg1* protein products: α‐SNAP_Rhg1_LC (from LC, Peking‐type *rhg1‐a*) and α‐SNAP_Rhg1_HC (from HC, PI 88788‐type *rhg1‐b*) (Cook et al., 2014; Figure 3a). The multicopy *G. soja rhg1‐ds* haplotypes and the product from *G. max* PI 342434 encoded an identical α‐SNAP that diverged from the susceptible *G. max* and *G. soja* reference genomes (wild‐type Wm82 and *G. soja* W05, and PI 483463, respectively), and from α‐SNAP_Rhg1_LC and α‐SNAP_Rhg1_HC (Figure 3a). To differentiate this protein from the much more common wild‐type α‐SNAP_Rhg1_ protein that is produced in *G. soja* accessions that carry single‐copy *Rhg1* haplotypes, and in adherence with the original naming convention for *Rhg1*‐encoded α‐SNAP proteins (Bayless et al., 2016), we termed this protein ‘α‐SNAP_Rhg1_Gsm’, for *G. soja* multicopy. The single‐ copy *Rhg1 G. soja* accessions studied because they carried the NSF_RAN07_ R_4_Q polymorphism were all found to encode an α‐SNAP amino acid sequence identical to that encoded in the SCN‐susceptible Williams 82 soybean reference genome. Like its counterparts in established SCN‐resistant soybean varieties, α‐SNAP_Rhg1_Gsm is polymorphic at amino acid residues predicted to form electrostatic interactions with NSF (Bayless et al., 2018). No other amino acid polymorphisms are present between the α‐SNAP of tested *Rhg1* single copy *G. soja* and α‐SNAP_Rhg1_Gsm. Curiously, some of the variant (non‐wild‐type) amino acid residues of α‐SNAP_Rhg1_Gsm at the C‐terminus are the same as in α‐SNAP_Rhg1_LC while others are the same as in α‐SNAP_Rhg1_HC. These shared residues again suggest, as with the shared *Rhg1* repeat junction and SNP‐based phylogeny, that there is a close evolutionary relationship between SCN‐resistant *G. max Rhg1* haplotypes and the *G. soja rhg1‐ds* haplotype.

α‐SNAP_Rhg1_Gsm was subsequently tested for two recently discovered resistance‐associated functions. Transient expression of *Rhg1*‐resistance type α‐SNAPs in *N. benthamiana* is cytotoxic and induces cell death by 6–7 d after infiltration with *A. tumefacien*s carrying this construct, with multiple lines of evidence suggesting that this phenotype is due to disruption of normal α‐SNAP–NSF function (Bayless et al., 2016, 2018). In the present experiments, introduction of a cDNA encoding α‐SNAP_Rhg1_Gsm driven by a constitutive promoter also caused obvious cell death in *N. benthamiana* leaves by 6 d after infiltration (Figure 3b). Expression of wild‐type α‐SNAP or an empty vector control did not cause any cell death, while expression of α‐SNAP_Rhg1_LC or α‐SNAP_Rhg1_HC caused death similar to that caused by α‐SNAP_Rhg1_Gsm (Figure 3b). Expression of all of the α‐SNAPs was confirmed using previously described custom antibodies (Figure 3c; Bayless et al., 2016, 2018).

Concurrent with cell death, overexpression of resistance‐type *Rhg1* α‐SNAPs has been found to cause elevated accumulation of endogenous NSF in *N. benthamiana*—an apparent cellular feedback response to disrupted NSF function (Barszczewski et al., 2008; Bayless et al., 2016; Naydenov et al., 2011; Zhao et al., 2007). Expression of α‐SNAP_Rhg1_Gsm caused similar accumulation of NSF, well above that observed in control leaves expressing either the α‐SNAP from Williams 82 or the empty vector (Figure 3c). These cytotoxicity and NSF accumulation results suggest that, like the widely used PI 88788‐type and Peking‐type *Rhg1* α‐SNAPs, the *G. soja* multicopy α‐SNAP_Rhg1_Gsm disrupts normal α‐SNAP–NSF function because of its C‐terminal polymorphisms.

### 3.4 *Glycine soja Rhg1* α‐SNAP interacts weakly with wild‐type NSF_Ch07_

In light of the above results, we investigated the interaction between α‐SNAP_Rhg1_Gsm and the two NSF types, wild‐type NSF (NSF_Ch07_) and *G. soja*‐encoded NSF_RAN07_. We first validated the presence of *NSF_RAN07_* in the six *G. soja rhg1‐ds* accessions using primers specific either to the *NSF_RAN07_* or *NSF_Ch07_* and *NSF_Ch13_* (*Glyma.13G180100*) as a positive control. As predicted by the initial SoySNP 50K screen of *G. soja* germplasm, all six of the tested accessions had *NSF_RAN07_* amplicons (Supplemental Figure S3). Interestingly, PI 507614B and PI 407287 gave amplicons for both *NSF_RAN07_* and *NSF_Ch07_* across plant samples, whereas the other accessions only gave amplicons to *NSF_RAN07_* (Supplemental Figure S3). Subsequent sequencing of PCR products from PI 507614B or PI 407287, generated using primers specific to the 5′ portion of *Glyma.07G195900* (5′ UTR and first intron), only showed sequences analogous to *NSF_RAN07_*. This suggests that an additional *NSF_Ch07_*–like locus may be adjacent to sequences in the genome not captured by our primer set. Cloning of *Glyma.07G195900* for each of the six *rhg1‐ds G. soja* accessions as well as PI 342434 *G. max* revealed a DNA sequence identical to that of the *NSF_RAN07_* from known resistant‐type soybeans. Sequencing of a PCR product carrying the 5′ portion of PI 468396B, previously characterized by from Zhang et al. (2017a), which does not have the R_4_Q polymorphism‐associated SNP, showed an *NSF_Ch07_* allele. This further indicates the accuracy of the above primer set. We subsequently determined the sequence of the *NSF_Ch07_* amplicons in PI 507614B and PI 407287, by cloning the amplicon into a pGEM‐T plasmid and sequencing. Intronic polymorphisms revealed that the amplicons derived from PI 507614B and PI 407287 have a sequence similar to the *NSF_Ch07_* from Williams 82. This suggests that there is an *NSF_Ch07_* allele in these accessions, although perhaps at a chromosomal location that was not amplified from the above primer set.

**FIGURE 4:**
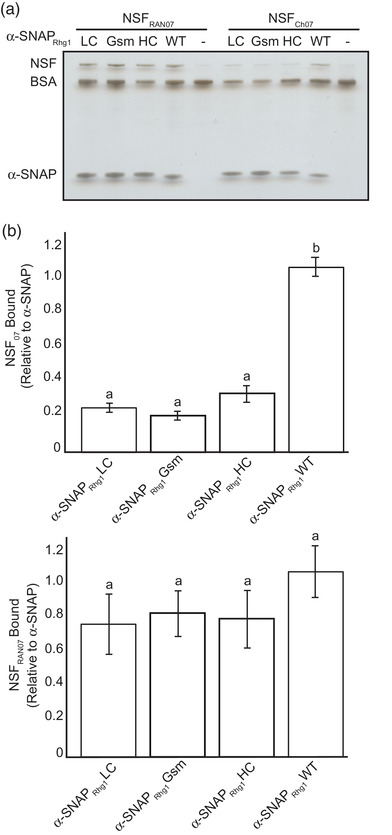
*Glycine soja rhg1‐ds* alpha‐Soluble NSF Attachment Protein (α‐SNAP_Rhg1_Gsm) interacts well with NSF_RAN07_ N‐ethylmaleimide Sensitive Factor protein but poorly with wild‐type NSF. (a) Representative silver‐stained SDS/PAGE gel of recombinant soybean wild‐type (WT) NSF_Ch07_ or NSF_RAN07_ bound in vitro by different recombinant α‐SNAP_Rhg1_ proteins in relative binding affinity assay. LC, α‐SNAP_Rhg1_LC (low copy); Gsm, α‐SNAP_Rhg1_Gsm; HC, α‐SNAP_Rhg1_HC (high copy); WT, Wild type (single‐copy) α‐SNAP_Rhg1_; BSA, bovine serum albumin (carrier protein). (b) Densitometric quantification of relative amount of NSF_Ch07_ (upper graph) or NSF_RAN07_ (lower graph) bound by the indicated α‐SNAP, collecting results from three independent experiments. Error bars show standard error of the mean. Bars with same letter are not statistically different (ANOVA, Tukey HSD *p* > .05)

We then proceeded to investigate physical interaction between α‐SNAP_Rhg1_Gsm protein and the two NSF types. Using an *Escherichia coli* expression system, we produced recombinant α‐SNAP_Rhg1_Gsm protein and NSF_Ch07_ and NSF_RAN07_ proteins, as well as α‐SNAP_Rhg1_LC and α‐SNAP_Rhg1_HC from resistant *G. max* varieties, and α‐SNAP_Rhg1_WT from single‐copy susceptible Williams 82. We then performed in vitro binding assays between the different NSFs and α‐SNAPs. Relative to single‐copy susceptible Williams 82, the resistance‐associated *G. max* α‐SNAPs interacted poorly with wild‐type NSF_Ch07_ (Figure 4a), recapitulating prior observations (Bayless et al., 2016, 2018). The *G. soja* α‐SNAP_Rhg1_Gsm also exhibited deficient interaction with wild‐type NSF_Ch07_ (Figure 4a). This suggests that, as occurs with the resistance‐associated soybean *Rhg1* α‐SNAPs, presence of the *G.soja* α‐SNAP_Rhg1_Gsm is likely to disrupt the normal cellular vesicle trafficking that requires efficient α‐SNAP–NSF interaction in the absence of NSF_RAN07_. These results are quantified in Figure 4b. We also investigated if α‐SNAP_Rhg1_Gsm interacts better with NSF_RAN07_ than with NSF_Ch07_. Consistent with previous findings regarding α‐SNAP_Rhg1_LC and α‐SNAP_Rhg1_HC (Bayless et al., 2018), there was marked increase in the binding of α‐SNAP_Rhg1_Gsm to NSF_RAN07_ relative to binding with wild‐type NSF_Ch07_ (Figures 4a and 4b).

### 3.5 *Glycine soja* with multicopy *rhg1‐ds* do not carry the *rhg1‐a* α‐SNAP copia retrotransposon, chromosome 11 α‐SNAP truncation, or resistance‐associated *Rhg4*

The additional genetic features known to most prominently associate with some *Rhg1*‐mediated SCN resistance are the *rhg1‐a* α‐SNAP copia (RAC) retrotransposon, the chromosome 11 α‐SNAP intron‐retention allele that encodes a nonfunctional truncated α‐SNAP, and resistance‐associated alleles of *Rhg4* that encode a serine hydroxymethyltransferase (Bayless et al., 2018, 2019; Lakhssassi et al., 2017; Liu et al., 2012). We examined the six *rhg1‐ds G. soja* accessions (and PI 342434 *G. max*) for these genetic features. None of these seven accessions had a SNP signature of RAC integration (ss715606985 G to A; Supplemental Table S1) or any PCR‐amplification features of the 4.8 kb RAC insertion at *Glyma.18G022500* (Figure S4b). They also did not carry short sequence repeats that could be a hallmark of transposon excision at a possible excised RAC site in *rhg1‐ds*.

**FIGURE 5:**
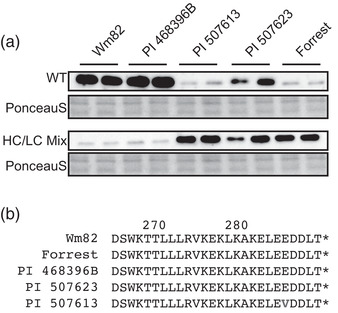
PI 507613 encodes a unique alpha Soluble NSF Attachment Protein on chromosome 11 (α‐SNAP_Ch11_). (a) Immunoblots of root protein extracts in *Resistance to Heterodera glycines 1* (*Rhg1*) *rhg1‐ds*‐containing *Glycine soja* and controls shows low apparent expression of α‐SNAP_WT_ in PI 507613. Paired lanes are replicate samples from same accession. Wild type (WT) and high copy–low copy (HC‐LC) mix refer to the polyclonal antibody used for detection. (b) Alignment of C‐terminal amino acids of α‐SNAP_Ch11_ in PI 507613 showing the E_285_V polymorphism, relative to other *rhg1‐ds*‐containing *G. soja* (PI 507623), single‐copy *G. soja* (PI 468396B), and *G. max* cultivars Williams 82 and Forrest

The genomic region that in some soybean accessions encodes an intron‐retention allele at the *Glyma.11G234500* α‐SNAP_Ch11_ gene was also sequenced. We first examined SoySNP50K data for the SNP associated with the intron retention allele ss715606985 C to T but noted that for many *G. soja* the nucleotide was uncalled at this SNP position. By PCR and sequencing, we determined that all seven of the *rhg1‐ds* accessions carried an allele lacking the intron retention SNP (Supplemental Figure S5). When antibodies against α‐SNAP_WT_ were used in protein immunoblots, we discovered that PI 507613, but not PI 507623, had a low apparent level of α‐SNAP_WT_ protein in both roots and shoots (Figure 5a; Supplemental Figure S6a). This low level of α‐SNAP_WT_ is analogous to previous experiments with *rhg1‐a* soybean lines that carry the α‐SNAP intron‐retention allele on chromosome 11 (Bayless et al., 2018). DNA sequencing revealed that while the encoded C‐terminus of α‐SNAP_Ch11_ in PI 507623 is similar to Williams82, the C‐terminus of α‐SNAP_Ch11_ in PI 507613 carries a single amino acid polymorphism (E_285_V; Figure 5b). The apparent low levels of α‐SNAP_WT_ protein in PI 507613 may be due to reduced recognition by the antibody used, or to other regulatory mechanisms, but apparently are not due to a *Glyma.11G234500* intron retention allele.

We also performed immunoblots with leaf and root extracts of PI 507613 and PI 507623 using other antibodies to detect resistance‐associated α‐SNAP_Rhg1_LC and α‐SNAP_Rhg1_HC. For tissue from developing trifoliates, immunoblots revealed a low basal expression of α‐SNAP_Rhg1_Gsm in *G. soja* PI 507613 and PI 507623 relative to the Forrest (*rhg1‐a*) control (Supplemental Figure S6a). However, in *rhg1‐ds* roots we observed more α‐SNAP_Rhg1_Gsm protein, at levels analogous to Forrest soybean roots (Figure 5a). Control blots of purified α‐SNAP_Rhg1_Gsm protein showed antibody recognition at sensitivities similar to that for α‐SNAP_Rhg1_HC or LC, against which the antibodies were raised (Supplemental Figure S6b).

*Rhg4* encodes a variant serine hydroxymethyltransferase in the allele (not genetically linked to *Rhg1*) that plant breeders pair with *rhg1‐a* to achieve useful SCN resistance in domesticated soybean (Liu et al., 2012; Patil et al., 2019; Yu et al., 2016). Sequencing of *Rhg4* cDNA clones from the six *rhg1‐ds G. soja* accessions (as well as soybean *Rhg4* transcripts from SCN‐susceptible cultivar Essex and SCN‐resistant Peking as controls) revealed absence of the P_130_R and N_358_Y polymorphisms associated with Peking‐type resistance, suggesting that the six *rhg1‐ds G. soja* accessions carry a susceptible‐type (wild‐type) *Rhg4*.

### 3.6 The *Glycine soja* PI 507613 and PI 507623 are resistant to virulent nematode populations

In light of the above observations about *rhg1‐ds*, including observations about the absence of a resistance‐associated *Rhg4* and chromosome 11 α‐SNAP alleles, we sought to determine the SCN resistance of select *rhg1‐ds G. soja* accessions. The *rhg1‐ds G. soja* lines PI 507613, PI 507623, and the *G. soja*‐like PI 342434 were tested with HG type 0 and HG type 2.5.7 nematode populations. We compared the FIs (relative number of cysts formed) to those for *G. max* HG type indicator lines and the *G. soja* PI 468916, which is the source of the SCN resistance QTL *cqSCN‐006* and *cqSCN‐007* (Wang et al., 2001) (Table 1). As might be predicted because of the absence of resistance‐associated *Rhg4* or chromosome 11 α‐SNAP alleles, PI 342434 was only able to confer moderate resistance to an HG type 0 SCN population. Intriguingly, PI 342434 was not able to confer substantial resistance to an HG type 2.5.7 SCN population. Even more intriguing, PI 507613 and PI 507623 were both able to confer strong resistance to HG type 0 and HG type 2.5.7 nematode populations, with levels comparable to PI 468916 (Table 1). The above findings, taken as a whole, do not demonstrate causation but do suggest that the novel *rhg1‐ds* haplotype is as functional as *rhg1‐a* for SCN resistance against HG type 0 SCN. The resistance of PI 507613 and PI 507623 to the HG type 2.5.7 SCN population (that PI 342434 was susceptible to) indicates that in PI 507613 and PI 507623, loci other than *rhg1‐ds* also contribute to SCN resistance against economically important HG type 2.5.7 SCN populations. A summary of these data may be found in Supplemental Table S2.

**TABLE 1:** Relative soybean cyst nematode (SCN) cyst production on select *Glycine soja* accessions and *Heterodera glycines* (HG) type indicator *G. max lines* for two nematode populations

		Nematode population	
HG type indicator line number	Accession	HG Type 0	HG Type 2.5.7
		Female index^a^	
1	‘Peking’	0	0
2	PI 88788	1	42
3	PI 90763	0	0
4	PI 437654	0	0
5	PI 209332	1	47
6	PI 89772	0	0
7	‘Cloud’	3	62
–	PI 507623	9	8
–	PI 507613	3	14
–	PI 342434	26	60
–	PI 468916	2	18

^a^ Female index is percentage of SCN cysts per root system, relative to the susceptible control (‘Lee 74') within the same experiment, for 10 plants per genotype.

### 3.7 *rhg1‐b* and *rhg1‐ds* made statistically similar partial‐resistance contributions against an HG type 2.5.7 SCN population

To test the effect of the novel *rhg1‐ds* allele on resistance relative to the *rhg1‐b* allele, a F_2_ population from a cross of *G. max* LD10‐10198 (*rhg1‐b* resistance) × *G. soja* PI 507623 (*rhg1‐ds*) was tested for SCN resistance. Each plant in the population was evaluated for resistance in a greenhouse using an HG type 2.5.7 SCN population against which *rhg1‐b* soybean exhibit partial resistance, and each plant was tested with the marker Satt309 that maps within 1 cM of *Rhg1* (Kim et al., 2010). One month after the plants were inoculated, the susceptible check Lee 74 averaged 219 SCN females, while the 96 F_2_ plants averaged 120 females. Within the F_2_ population, no significant association was observed between the Satt309 allele present and level of SCN resistance [single factor ANOVA Pr(>*F*) = 0.34]. Hence *rhg1‐ds* was associated with partial resistance—no distinction in their contribution to resistance against the tested HG type 2.5.7 SCN population was detected between *rhg1‐b* and the *rhg1‐ds* haplotype derived from PI 507623. This experiment, together with that of Table 1 (above), also provides further evidence that there are loci other than *Rhg1* in PI 507623 that contribute to the observed high‐level resistance of PI 507623 to the virulent HG type 2.5.7 SCN population that was used.

## 4 DISCUSSION

Crop wild relatives are an appealing source for novel traits of agronomic interest, and this is evident in domesticated soybean. Recent resequencing of *G. soja* along with landrace and elite *G. max* varieties revealed that many rare alleles were lost from *G. soja* during its domestication to form *G. max* (Hyten et al., 2006; Liu et al., 2020). Many of these lost traits are of agronomic importance. Quantitative trait loci associated with improved yield, salt tolerance, alkaline salt tolerance, soybean aphid resistance, foxglove aphid resistance, and SCN resistance have all been found to be present in *G. soja* but absent in *G. max* (Concibido et al., 2003; Hesler et al., 2013; Kabelka et al., 2005, 1988; Kim et al., 2013; Lee et al., 2009, 2015a; Wang et al., 2001; Yu & Diers, 2017). With respect to SCN, *G. soja* PI 468916 has served as a donor for the QTL *cqSCN‐006* and *cqSCN‐007* that each contribute modest resistance to a highly virulent SCN population but provide substantial and synergistic resistance even to HG type 1.2.3.5.6.7 SCN when combined in *G. max* with *rhg1‐b* and a chromosome 10 QTL from PI 567516C (Brzostowski et al., 2017). Here, we used a SNP marker associated with *NSF_RAN07_* to select *G. soja* accessions to screen for multicopy *Rhg1* haplotypes, leading to discovery of the *rhg1‐ds* haplotype that encodes a unique α‐SNAP with SCN resistance‐associated functional features. The hypothesis was that plant germplasm accessions that are strong candidates to carry alleles of interest can be identified using available SNP genotypes for particular alleles of other (unlinked) genes that also contribute to the trait of interest. Our study highlights the power of deploying new findings regarding the genetic architecture of traits of interest (such as the requirement for NSF_RAN07_ in *Rhg1*‐mediated SCN resistance), along with available data such as the SoySNP50K data for ∼20,000 *Glycine* accessions, to prescreen germplasm and identify a manageable number of accessions for functional analysis. Recent next‐generation sequencing work has further identified structural and nucleotide variants within domesticated soybean (Torkamaneh et al., 2019). The same study also identified loss of function alleles within soybean germplasm, potentially functioning as a sequence‐catalogued mutant library analogous to those available in *Arabidopsis thaliana* (L.) Heynh. If genetic features of potential interest are known, there are increasingly powerful resources available for in silico germplasm prescreens.

A potential limitation of using the NSF_RAN07_ SNP for in silico germplasm screens is that it may not fully capture the diversity of *Rhg1*‐containing *G. soja*. There may be lines that do not share with soybean (Bayless et al., 2018) the requirement for NSF_RAN07_ for *Rhg1*‐mediated SCN resistance. In the present study and in previous work, NSF_RAN07_ was found to be present in all multicopy *Rhg1* accessions but also in a minority of the much larger pool of single‐copy *Rhg1* accessions (Bayless et al., 2018), suggesting that NSF_RAN07_ is necessary for multicopy *Rhg1* resistance while demonstrating that NSF_RAN07_ can be present in the absence of multicopy *Rhg1* resistance. Some accessions expressing NSF_RAN07_ but lacking multicopy *Rhg1* might exhibit elevated SCN resistance, but that trend has not been observed in the documented SCN resistance of soybean accessions that express NSF_RAN07_ but lack multicopy *Rhg1*.

We and others have identified SoySNP 50K (or KASP) SNPs associated with other resistance‐associated genetic elements that might be used during in silico germplasm screens for possible SCN resistance. In addition to NSF_RAN07_, these include the chromosome 11 intron retention α‐SNAP knockout, the RAC (*rhg1‐a*‐alpha SNAP copia) retrotransposon in the first intron of *rhg1‐a* α‐SNAP genes, and SNPs specific for SCN resistance‐associated alleles of *Rhg4* (Bayless et al., 2018, 2019; Matsye et al., 2012; Shi et al., 2015; Tran et al., 2019). These might be used to select *G. soja* accessions that can subsequently be tested for the presence of useful alleles at other loci that contribute to SCN resistance whose presence is not adequately tested by the SoySNP50K dataset. More direct tests using markers not on the SoySNP50K chip could also be carried out de novo on large numbers of germplasm accessions (albeit at appreciable new expense), for example to detect *Rhg1* tandem repeat junctions, novel C‐terminal sequences in α‐SNAP proteins, or new *Rhg4* alleles.

The present discovery of PI 507613 as an accession of interest for SCN resistance provides an extended example of the above hypothesis, that useful alleles at multiple loci controlling a trait are likely to co‐occur in individual accessions. Phenotypic screening of USDA accessions for SCN resistance apparently missed PI 507613, which was instead flagged for further study by our *NSF_RAN07_* genotypic screen. PI 507613 would have been passed over in a screen for RAC but would be positive in a screen for the *Rhg1* tandem repeat junction. PI 507613 would also be negative for *Rhg4* or the chromosome 11 intron‐retention α‐SNAP, but we did discover another α‐SNAP with unusual C‐terminal amino acids in this accession. The example gains more interest when considering that, in addition to finding the novel *rhg1‐ds*, we found that PI507613 carries the valuable trait of SCN resistance to HG type 2.5.7 SCN (that apparently is not dependent on traditional *rhg1‐a*/*Rhg4*/chromosome 11 α‐SNAP genotypes). This PI 507613 SCN resistance is dependent on more loci than just *rhg1‐ds*. We postulate that it was much more likely to identify such an accession once we focused on *G. soja* accessions that carry one or more SCN resistance alleles of interest. A productive target for future studies will be to map and identify the full complement of other loci that contribute to resistance to HG type 2.5.7 SCN in PI 507613, PI 507623, and possibly in the other *rhg1‐ds* PIs identified in this study.

Unlike domesticated soybean, *G. soja* accessions are not known to contain the resistance‐associated allele of *Rhg4*, which is necessary for the efficacy of SCN resistance conferred by *rhg1‐a* in *G. max* (Brucker et al., 2005; Meksem et al., 2001; Wu et al., 2016; Yu et al., 2016). Our findings are consistent in these observations. The accessions we examined did not have a resistance‐type *Rhg4* or other attributes of Peking‐type SCN resistance: presence of RAC or the nonfunctional chromosome 11 α‐SNAP allele (although in PI 507613 there does seem to be a novel α‐SNAP_Ch11_). Previous GWAS studies with *G. soja* have either not detected significant contributions from the *Rhg1* locus when testing with a Race 1 (HG type 2.5.7) SCN population (Zhang et al., 2017a) or detected only a minor contribution (*R* ^2^ = 13.6%) from a locus 5–12 Mb (>8 genes) away from *Rhg1* when testing with a separate HG type 2.5.7 SCN population (Zhang et al., 2016). Those GWAS results may have been impacted by a very low rate of occurrence of multicopy *Rhg1* loci in *G. soja*. However, they are also consistent with poor expression of the SCN resistance phenotype that might be predicted if a haplotype such as *rhg1‐ds* is not coupled with complementary *Rhg4*/*RAC*/chr11 α‐SNAP genotypes. This makes it all the more interesting to understand the other genetic features that contribute to any observed SCN resistance in *G. soja* (possibly including loci identified by Kabelka et al [2005], Winter et al. [2007], Arelli et al. [2000], Vuong et al. [2015], Zhang et al. [2017a, 2016, 2017b], and other studies). Presumably, novel cellular mechanisms that could enhance the durability of SCN resistance are encoded at those loci.

The evolutionary history of the economically important *rhg1‐b* and *rhg1‐a* soybean loci is a point of interest, and not only because of the unique structure of these loci (copy number variation of up to 10 copies of a four gene chromosomal block, in which three of the four tightly linked genes contribute to SCN resistance). Investigations of *Rhg1* evolution may also reveal new *Rhg1* haplotypes or suggest approaches to additional functional engineering of *Rhg1* components. The previous cloning and characterization of both *rhg1‐a* and *rhg1‐b* demonstrated that the bridge junction (the junction between two *Rhg1* blocks) is conserved (Cook et al., 2012). This suggested that *Rhg1* duplication preceded the split between *rhg1‐a* and *rhg1‐b*. Further studies have revealed an array of copy numbers for *rhg1‐a* and *rhg1‐b* haplotypes in various soybean accessions (Cook et al., 2014, 2012; Lee et al., 2016, 2015b; Patil et al., 2019; Yu et al., 2016). Whole‐genome resequencing had suggested the existence of a *rhg1‐a‐*like *Rhg1* within *G. soja*, providing evidence for an early origin of *Rhg1* outside of *G. max* (Lee et al., 2015b). The data we present provides more complete evidence of an early origin of multicopy *Rhg1*, and revealed a novel haplotype and novel α‐SNAP_Rhg1_. Further, this study identified *rhg1‐ds* as a candidate progenitor of *rhg1‐a* and *rhg1‐b* and α‐SNAP_Rhg1_Gsm as a candidate progenitor of α‐SNAP_Rhg1_LC and α‐SNAP_Rhg1_HC. The multiyear undertaking has been initiated to generate transgenic soybean lines in which isogenic presence or absence of the allele encoding α‐SNAP_Rhg1_Gsm can be associated with resistance to different HG types of SCN.

Vesicle trafficking is essential to eukaryotic cellular homeostasis. The aberrant α‐SNAP encoded by resistance‐associated *Rhg1* haplotypes has been implicated in conferring resistance to SCN (Bayless et al., 2016; Cook et al., 2014, 2012; Lakhssassi et al., 2017, 2020; Liu et al., 2017). Interestingly, the only SNPs causing nonsynonymous codon differences in the *Rhg1* proteins from resistant and susceptible cultivars occur within the gene body of the α‐SNAP, particularly at the C‐terminus—the region that stimulates NSF activity for SNARE disassembly (Barnard et al., 1997; Bayless et al., 2016; Cook et al., 2014; Marz et al., 2003; Zhao et al., 2015). Here, we report an additional resistance‐associated α‐SNAP with a C‐terminus distinct from that of either known resistant types or the standard wild‐type α‐SNAP. Variation in nematode functions that interface with host plant α‐SNAPs might be associated with SCN virulence. Recent work elucidating effectors of SCN suggests that SNARE‐like proteins—proteins that normally interact with α‐SNAP and NSF to form the 20S complex—constitute a large and variable effector family in SCN (Gardner et al., 2018). Further, there is some molecular evidence for direct physical interaction between SNARE‐like protein effectors and α‐SNAP with virulence outcomes (Bekal et al., 2015). Moreover, recent evidence suggests a potential physical interaction between the *Rhg4*‐encoded serine hydroxymethyltransferase and the α‐SNAP_Rhg1_, as well as α‐SNAP_Rhg1_ and syntaxin proteins, both with implications for resistance (Dong et al., 2020; Lakhssassi et al., 2020). In light of this, varying the soybean α‐SNAP repertoire (for example by use of *rhg1‐ds* and α‐SNAP_Rhg1_Gsm) may hinder the capacity of nematodes to overcome *Rhg1*‐mediated resistance.

The present study also discovered a novel α‐SNAP on chromosome 11 in PI 507613. From previous work, it is apparent that SCN resistance involves not just presence of a α‐SNAP_Rhg1_ but also modification of the relative amount of wild‐type α‐SNAP protein in syncytia or in whole plants (Bayless et al., 2016, 2018, 2019). It is possible that the novel α‐SNAP_Ch11_ of PI 507613 is a bona fide resistance‐associated QTL. Indeed, under a model where HG type 0 or HG type 2.5.7 nematode populations produce effectors that interact with α‐SNAP_WT_ toward facilitating infection or reproduction, modifying the α‐SNAP_WT_ could change virulence outcomes as a result of reduced interaction. Future work should be directed toward understanding whether the novel α‐SNAP_Ch11_ cosegregates with SCN resistance.

It may be possible to engineer soybean with novel α‐SNAPs by genetic modification and gene‐editing methods, or by classical breeding to bring in different α‐SNAPs such as the *G. soja rhg1‐ds* product. Through work such as that of Marz et al. (2003), a library of mammalian α‐SNAP mutations and known consequences is already available, and this type of structure–function knowledge (see also Zhao et al., 2015) is ready to be translated to soybean α‐SNAP. Vesicle trafficking has been implicated in many plant‐pathogen interactions beyond SCN (Hoefle & Hückelhoven, 2008; Inada & Ueda, 2014). Because of the seeming centrality of vesicle trafficking in plant defense, α‐SNAP protein sequences and expression patterns may also represent appealing targets to modify in order to confer resistance to other biotrophic pathogens.

## AUTHOR CONTRIBUTIONS

Derrick G. Grunwald: Conceptualization; Investigation; Writing‐original draft; Writing‐review & editing. Ryan W. Zapotocny: Conceptualization; Investigation; Writing‐review & editing. Seda Ozer: Investigation; Writing‐review & editing. Brian Diers: Funding acquisition; Investigation; Writing‐review & editing. Andrew Bent: Conceptualization; Funding acquisition; Investigation; Writing‐original draft; Writing‐review & editing.

## CONFLICT OF INTEREST

The authors declare that they have no competing interests.

## Supporting information

Supplemental Table S1: Single nucleotide polymorphism identity of select *Glycine max* and *Glycine soja* accessions
Supplemental Table S2: *Rhg1* copy number and relevant a‐SNAP, RAC, and NSF alleles present in domesticated soybean or select *G. soja* characterized in this study as they relate to SCN resistance
Supplemental Table S3. List of oligonucleotide primers used in this study
Supplemental Figure S1. Validation of *Resistance to Heterodera glycines 1* (*Rhg1*) repeat junction and multi‐copy *Rhg1* within *Glycine soja*.
Supplemental Figure S2. *rhg1‐ds*‐containing G*lycine soja* form a sub‐group within *G. soja* and *G. max*.
Supplemental Figure S3. Multicopy *rhg1‐ds Glycine soja* contain NSFRAN07.
Supplemental Figure S4. *rhg1*‐*ds*‐containing *Glycine soja* lack the RAC *rhg1‐a*‐associated retrotransposon.
Supplemental Figure S5. Multicopy *rhg1*‐*ds*‐containing *Glycine soja* do not contain the chromosome 11 α‐SNAPCh11 intron retention allele.
Supplemental Figure S6. Some *rhg1‐ds*‐containing G*lycine soja* have lower apparent expression of multicopy‐encoded α‐SNAPRhg1Gsm and/or wild‐type α‐SNAP in developing trifoliates.

## Data Availability

The data supporting the conclusions of this article are included within the article and the supplementary materials.
