# Supplementary material for: Detection of rare nematode resistance Rhg1 haplotypes in Glycine soja and a novel Rhg1 α‐SNAP
Source: Plant Genome. 2021 Oct 30;15(1):e20152. doi: 10.1002/tpg2.20152 (PMC12807349; doi:10.1002/tpg2.20152)
Supplement: Supplementary file 1 [file TPG2-15-e20152-s001.pdf]

Supplemental Materials - Grunwald *et al.* 2021 - Novel *G. soja Rhg1*

Table S1: Single nucleotide polymorphism identity of select *Glycine max* and *Glycine soja* accessions[1]

Accession	ss715597431 (NSF$_{RAN07}$) SNP identity	ss715610416 (α-SNAP$_{Ch11}$-IR) SNP identity[2]	ss715606985 (RAC) SNP identity
PI 342434	T	C	G
PI 378695B	U	U	G
PI 366121	T	U	G
PI 378695A	T	U	G
PI 407260	T	U	G
PI 407287	T	U	G
PI 504290	T	U	G
PI 507580	T	U	G
PI 507600	T	U	G
PI 507601A	T	U	G
PI 507602	T	U	G
PI 507603	T	U	G
PI 507604	T	U	G
PI 507608	T	U	G
PI 507613	T	U	G
PI 507614B	T	C	G
PI 507623	T	U	G
PI 549047	T	U	G
PI 597454A	T	U	G
PI 597458C	T	U	G
PI 597461A	T	C	G
PI 597461C	T	U	G
PI 518671 (Wm82)	C	C	G
PI 88788	T	C	G
PI 548402 (Peking)	T	T	A

[1]*Glycine soja* accessions with single nucleotide polymorphism indicative of R_4Q NSF$_{RAN07}$ mutation are shown (21 out of 1169 *Glycine soja* accessions screened using SoySNP50K data), along with PI 342434, resistant and susceptible *G. max* (PI 88788 and PI 518671, respectively), and resistant *G. soja* (PI 548402) controls on last three lines. Non-functional intron-retention mutation (α-SNAP$_{Ch11}$-IR), and *Rhg1* α-SNAP Copia (RAC) SoySNP50K SNP genotypes shown in final two columns. U indicates uncalled SNP.

[2]See also Figure S5 for DNA sequence data obtained for same region.

Table S2: *Rhg1* copy number and relevant α-SNAP, RAC, and NSF alleles present in domesticated soybean or select *G. soja* characterized in this study as they relate to SCN resistance

Soybean Accession	*Rhg1* haplotype	*Rhg1* Copy Number	SCN Resistance		*Rhg1* α-SNAP C-terminal Sequence	RAC Element Presence	Chrom. 11 α-SNAP Intron Retention Presence	Chrom. 11 α-SNAP C-terminal Sequence	Chrom. 7 NSF
			HG type 0	HG type 2.5.7					
Williams 82	*rhg1-c*	1	No	No	-EDDLT	No	No	-EDDLT	WT
Peking	*rhg1-a*	3	Yes	Yes	-YEVIT	Yes	Yes	-EDDLT	RAN07
Fayette	*rhg1-b*	10	Yes	No	-QHEAIT	No	No	-EDDLT	RAN07
PI 507613	*rhg1-ds*	~3	Yes	Yes	-HEVIT	No	No	-VDDLT	RAN07
PI 507623	*rhg1-ds*	~3	Yes	Yes	-HEVIT	No	No	-EDDLT	RAN07
PI 342434	*rhg1-ds*	~3	Moderate	No	-HEVIT	No	No	-EDDLT	RAN07

Table S3. List of oligonucleotide primers used in this study

5' Rhg1 Fw: TGACTAAGCAGCAAGCACAGA
5' Rhg1 Rev: TCTTTCCGTGTACGGTCGTC
3' Rhg1 Fw: AGAATACGTGGAGGCACAGC
3' Rhg1Rev: CCATGCGTTACGATGCGATG
Bridge Fw-1: TTTAGCCTGCTCCTCACAAATTC
Bridge Rev-1: TTGGAGAATATGCTCTCGGTTGT
Bridge Fw-2: TTTGGGCCTTCCTCGAACA
Bridge Rev-2: CAGTGCATCAAGAGCATGCAA
5' RAC Fw: CGATAAAGCCGCCAATTGCT
5' RAC Rev: TCACCAAAGCTCTTGCACCT
3' RAC Fw: GGAGATGGCCACCGAATGAA
3' RAC Rev: GTATGTCGCTCCAGCCTTGT
RAC across Fw: CAATTGCTTCAAGCTCGCCA
RAC across Rev: GTATGTCGCTCCAGCCTTGT
RAC Gibson Fw: TTGTTGACTCGACAGAAAGCCGCCAATTGCTTCAA
RAC Gibson Rev: GGTCGAATTCGCCCTTTTCAAGCAATGTGCAGCATCGACA
a-SNAP$_{Ch11}$ Intron Gibson Fw: TTGTTGACTCGACAGGAGATAGCTCGCCAATCCC
a-SNAP$_{Ch11}$ Intron Gibson Rev:
GGTCGAATTCGCCCTTTTCACTTCAATCTGGCAAATATTTCCAT
a-SNAP$_{Ch11}$ last Exon Fw: GGGGAGGCAATAGCTTAC
a-SNAP$_{Ch11}$ last Exon Rev: CAAGATATAATTCAATTCATAGTGTAGAT
Rhg4 cDNA Fw: ATGGATCCAGTAAGCGTGTGG
Rhg4 cDNA Rev: CTAATCCTTGTACTTCATTTCAG
Bridge qRT Fw: GCCGTGACTTCTTAACAAATGCAGC
Bridge qRT Rev: TGGGTAGTTTTGTTTCTTGCTCCAC
2620 qRT Fw: AAGCCCAACAGGCCAAAGAGAG
2620 qRT Rev: ACACCAAATGGGTTCGCACTTC
a-SNAP$_{Rhg1}$ Last Intron Fw: CAAGGAATTTGATAGTATGACCCCTC
a-SNAP$_{Rhg1}$ 3' UTR Rev: AGATAAGATCAGACTCCAGCAACCTC
a-SNAP$_{Rhg1}$-Gsm pBS Gisbon Fw:
TTGTTGACTCGACAGATGGCCGATCAGTTATCGAAGGG
a-SNAP$_{Rhg1}$-Gsm pBS Gibson Rev:
AGCTGGGTCGAATTCGCCCTTTTCATCAAGTAATAACCTCATGCTCCTCAAGTTC
a-SNAP$_{Rhg1}$-Gsm pRham Gibson Fw:
CGCGAACAGATTGGAGGTGCCGATCAGTTATCGAAGGGAGAGG
a-SNAP$_{Rhg1}$-Gsm pRham Gibson Rev:
GCTCAGCGGTGGCGGCCGCTCTATTAAGTAATAACCTCATGCTCCTCAAGTTC
NSF$_{Ch07}$ Detect Fw: CAACACGCCCGCGAGCGAC
NSF$_{RAN07}$ Detect Fw: CTACACGCCCGCGAACGAC
NSF Detect Rev: CTCACTTGTACGGAATCACCGG
NSF$_{Ch13}$ Fw: GAAACAGAGAAACATAGAGGCCAT
NSF$_{Ch13}$ Rev: CATTCAACGCAATCTGGCCG
NSF$_{Ch07}$ 5' Genomic Gibson Fw:
TTGTTGACTCGACAGGCTCGTAAGTCGTGTTTATAGCC

Table S3, continued:

NSF_{Ch07} 5’ Genomic Gibson Rev:
GGTCGAATTCGCCCTTTTCAAGCCAGTAGAACAGCATCAAT
NSF_{Ch07} 3’ Genomic Fw: CTGCTGCAGAGGCGTTGAATG
NSF_{Ch07} 3’ Genomic Rev: ACCTCAATACGAGACGTACGTTTTGAT
NSF_{RAN07} cDNA pBS Gibson Fw: TTGTTGACTCGACAGATGGCGAGTCAGTTCGGG
NSF_{RAN07} cDNA pBS Gibson Rev:
GGTCGAATTCGCCCTTTTCATTATAACCTAACAACATCCTGGAGGC

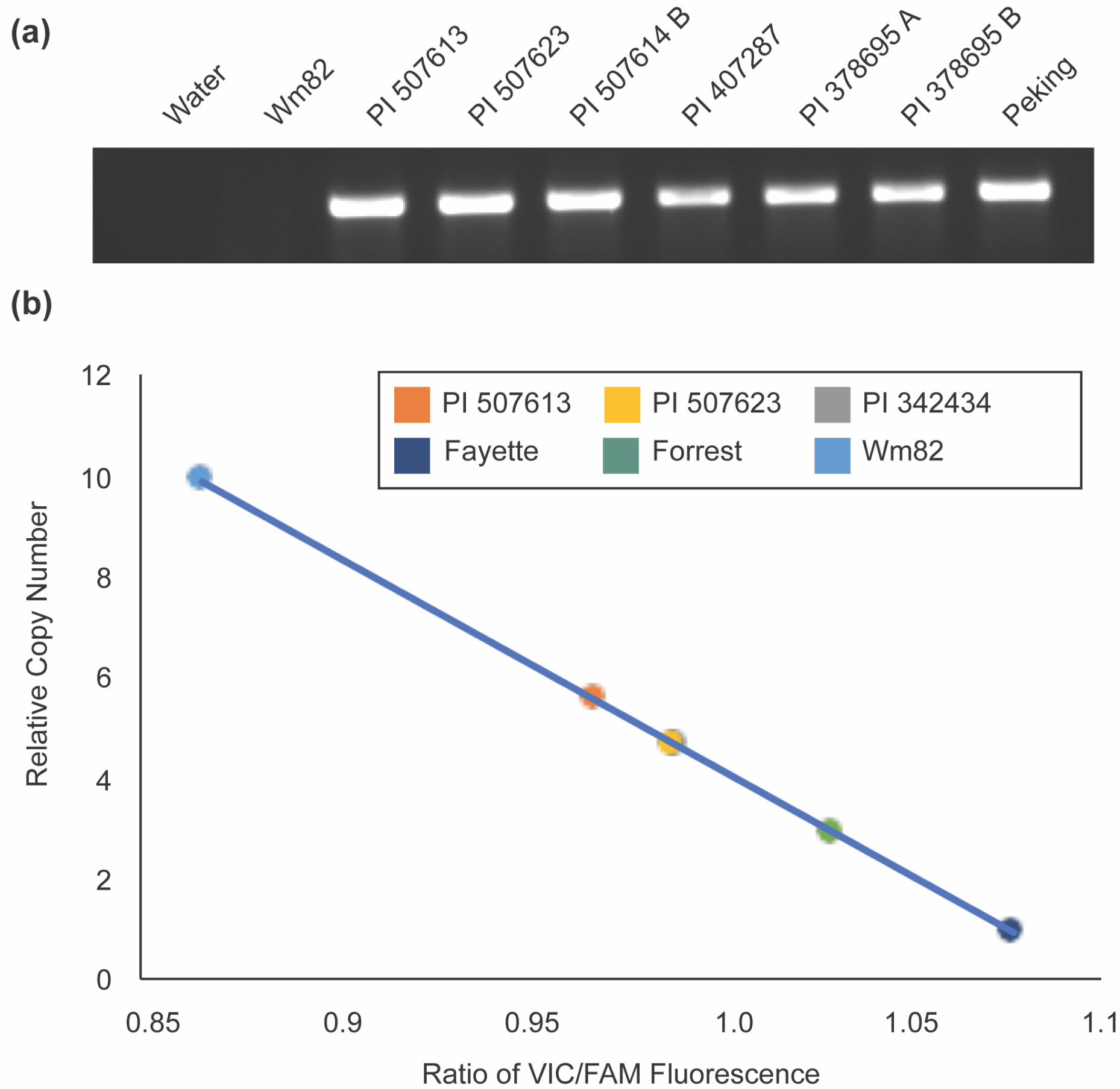

Figure S1. Validation of *Resistance to Heterodera glycines 1* (*Rhg1*) repeat junction and multi-copy *Rhg1* within *Glycine soja*. (a) Repeat junction was validated in a separate plant from those in Figure 1 using a different primer set. (b) Example of TaqMan assay on PI 507613, PI 507623, and PI 342434 along with *Glycine max* (Williams 82;Wm82), Fayette and Peking. Copy number estimated as the ratio of fluorescence of VIC (probe anneals *Rhg1* repeat) to FAM (probe anneals to single copy homeolog on chromosome 11). Blue line is the line of regression generated from Wm82 , Peking and Fayette (blue line) ratios. Orange=PI 507613, Grey=PI 342434, Yellow=PI 507623, Fayette=dark blue, Forrest=green, Wm82=cyan. Note however that highly variable results were obtained between separate experiments.

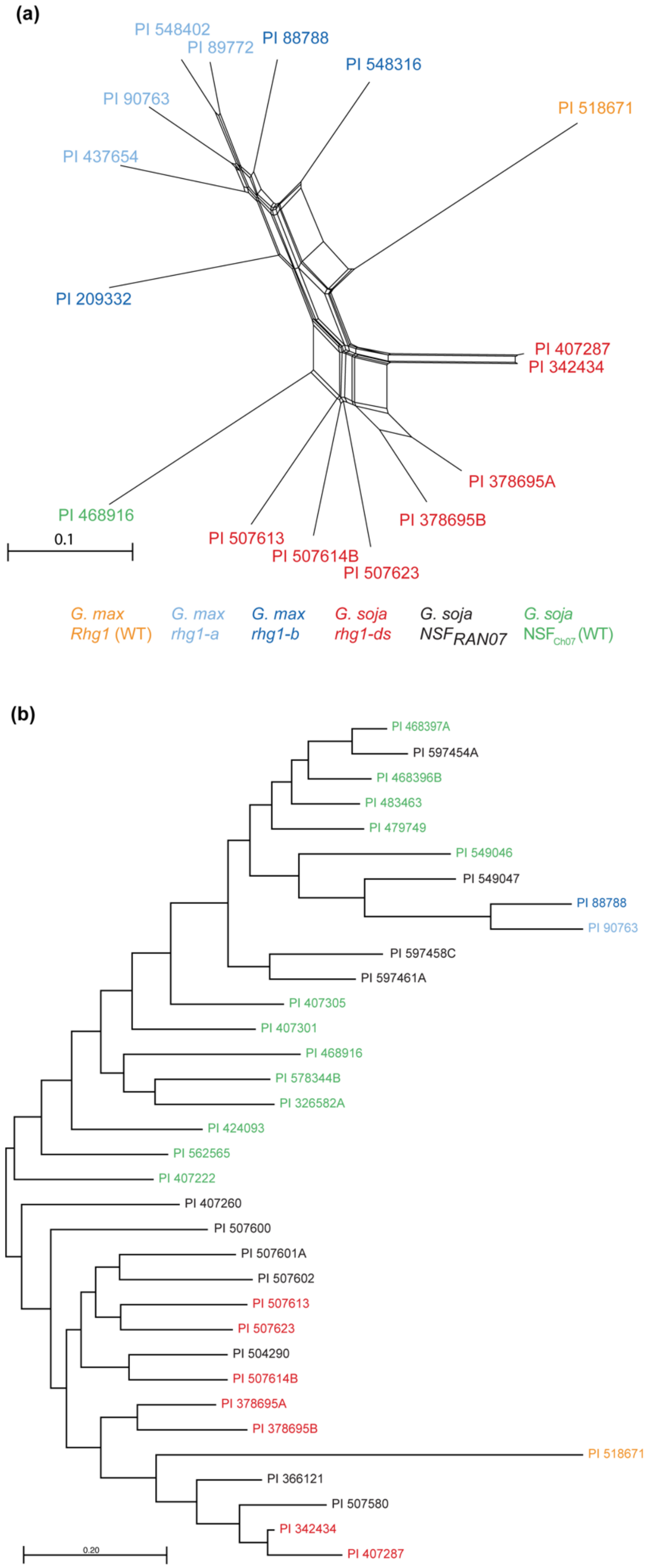
(a)
PI 548402
PI 89772
PI 88788
PI 548316
PI 90763
PI 518671
PI 437654
PI 209332
PI 407287
PI 342434
PI 378695A
PI 468916
PI 378695B
PI 507613
PI 507614B
PI 507623
0.1
G. max Rhg1 (WT)
G. max rhg1-a
G. max rhg1-b
G. soja rhg1-ds
G. soja NSF_{RAN07}
G. soja NSF_{Ch07} (WT)
(b)
PI 468397A
PI 597454A
PI 468396B
PI 483463
PI 479749
PI 549046
PI 549047
PI 88788
PI 90763
PI 597458C
PI 597461A
PI 407305
PI 407301
PI 468916
PI 578344B
PI 326582A
PI 424093
PI 562565
PI 407222
PI 407260
PI 507600
PI 507601A
PI 507602
PI 507613
PI 507623
PI 504290
PI 507614B
PI 378695A
PI 378695B
PI 518671
PI 366121
PI 507580
PI 342434
PI 407287
0.20

Figure S2. ***rhg1-ds*****-containing G*****lycine soja*** **form a sub-group within *G. soja* and *G. max*.**
(a) Unrooted phylogeny generated from whole-genome single nucleotide polymorphism data comparing *rhg1-ds*-containing *G. soja* (red) to *G. max* soybean cyst nematode indicator lines from Table 1 (*rhg1-a* in light blue and *rhg1-b* in dark blue). (b) Phylogenetic tree generated from whole-genome SNP data (SoySNP50K) comparing *G. soja* that carry *rhg1-ds* and NSF_{RAN07} (red) to previously published *G. soja* of agronomic interest, that do not carry either *rhg1-ds* or NSF_{RAN07} (green). NSF_{RAN07}-containing *G. soja* that lack *rhg1-ds* marked with black, and Williams 82 (WT) in orange.

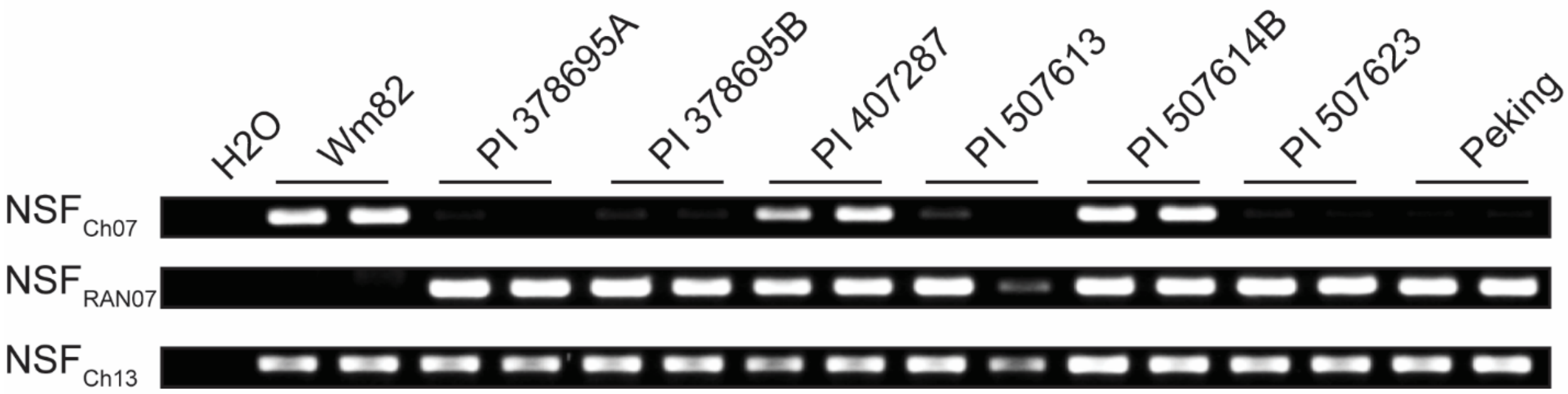

Figure S3. Multicopy *rhg1-ds Glycine soja* contain NSF$_{RAN07}$.

Presence of the R$_4$Q polymorphism is verified in *G. soja* containing multicopy *Rhg1* (middle) relative to the wild-type R$_4$ (NSF$_{Ch07}$; top), or to chromosome 13 NSF (bottom). Data generated using allele-specific primers (Table S3). Pairs of bands are replicate PCR reactions for separate genomic DNA samples from the same accession.

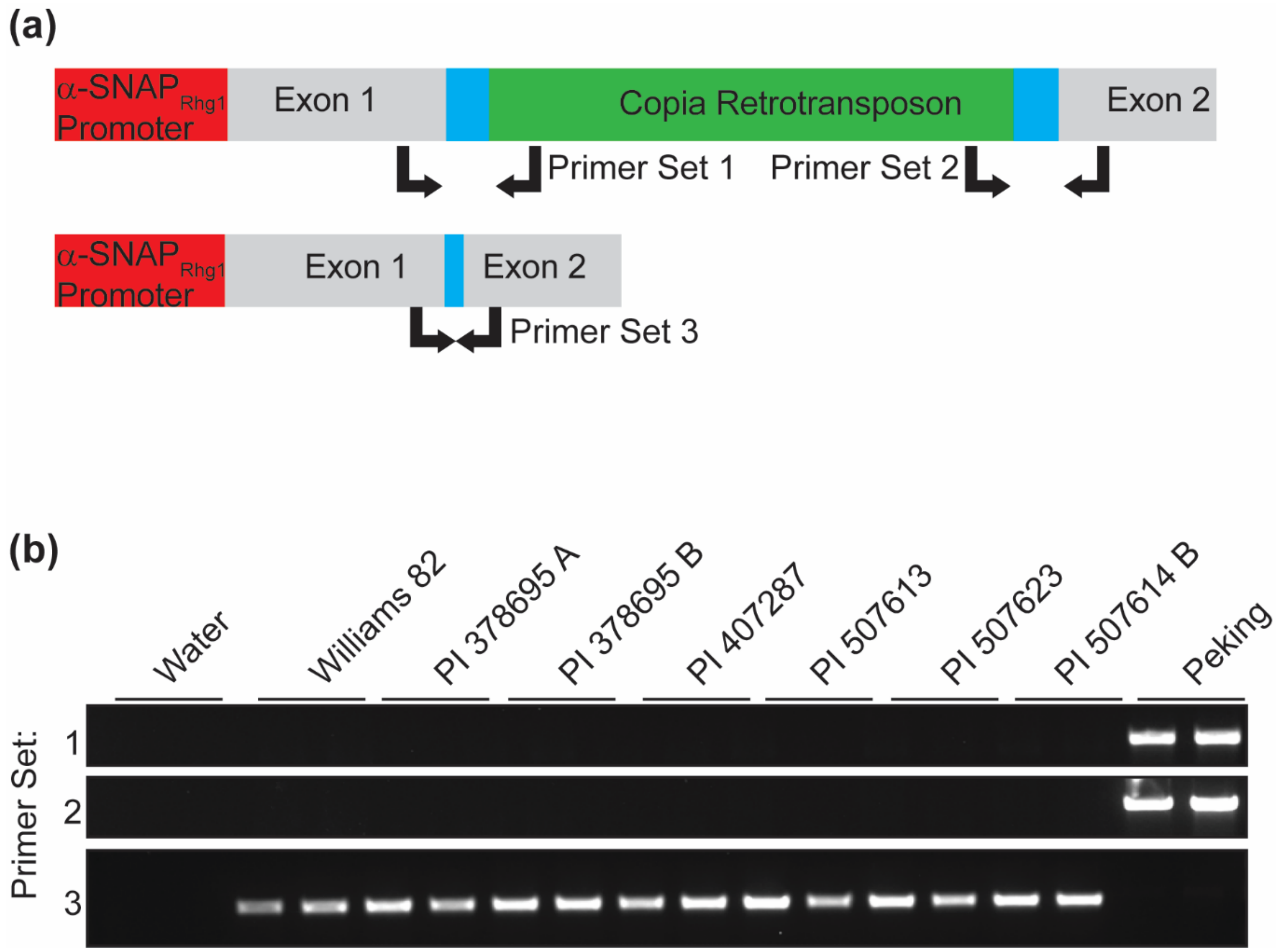

Figure S4. *rhg1-ds*-containing *Glycine soja* lack the RAC *rhg1-a*-associated retrotransposon. (a) Schematic of *Rhg1* α-SNAP copia (RAC) retrotransposon integration within known low-copy *Rhg1* soybean varieties. Primers used in (b) against the unique 5' and 3' junctions as well as across the entire first intron are also shown. (b) Gel showing amplification of the junctions as well as across the first intron in *G. max* cvs. Peking (a low-copy variety), Williams 82 (a single-copy variety) and the *rhg1-ds G. soja* accessions. Paired samples are replicate PCR reactions for different genomic DNA preparations of the same accession.

```
Wm82         AGATAGCTCGCCAATCCCTCAACAATAATTTGCTGAAGTATGGAGTTAAAGGACACCTGC  60
PI_507623    AGATAGCTCGCCAATCCCTCAACAATAATTTGCTGAAGTATGGAGTTAAAGGACACCTGC  60
PI_209332    AGATAGCTCGCCAATCCCTCAACAATAATTTGCTGAAGTATGGAGTTAAAGGACACCTGC  60
Peking       AGATAGCTCGCCAATCCCTCAACAATAATTTGCTGAAGTATGGAGTTAAAGGACACCTTC  60

Wm82         TTAATGCTGGCATCTGCCAACTCTGTAAAGGGGATGTTATTGCTGTAACCAATGCATTAG  120
PI_507623    TTAATGCTGGCATCTGCCAACTCTGTAAAGGGGATGTTATTGCTGTAACCAATGCATTAG  120
PI_209332    TTAATGCTGGCATCTGCCAACTCTGTAAAGGGGATGTTATTGCTGTAACCAATGCATTAG  120
Peking       TTAATGCTGGCATCTGCCAACTCTGTAAAGAGGAGGTTGTTGCTATAACCAATGCATTAG  120

Wm82         AACGATATCAGGTCTAAGTTTTTTCAATAGTCAACTTCTTGTGACTGGACAGCTTATTTG  180
PI_507623    AACGATATCAGGTCTAAGTTTTTTCAATAGTCAACTTCTTGTGACTGGACAGCTTATTTG  180
PI_209332    AACGATATCAGGTCTAAGTTTTTTCAATAGTCAACTTCTTGTGACTGGACAGCTTATTTG  180
Peking       AACGATATCAGGTCTAAGTTTTTTCAATAGTCAACTTCTTGTGACTGGACAGCTTATTTG  180

Wm82         TTGCTCAATTATTTAGATATGTTTTTATTTTGCAGGAACTGGATCCAACATTTTCGGGAA  240
PI_507623    TTGCTCAATTATTTAGATATGTTTTTATTTTGCAGGAACTGGATCCAACATTTTCAGGAA  240
PI_209332    TTGCTCAATTATTTAGATATGTTTTTATTTTGCAGGAACTGGATCCAACATTTTCGGGAA  240
Peking       TTGCTCAATTATTTAGATATGTTTTTATTTTGCAGGAACTGGATCCAACATTTTCAGGAA  240

Wm82         CACGTGAATATAGATTTTTGGCGGTAGGTCACTAGTTTTGGTATTTCGTTATTATTTTTT  300
PI_507623    CACGTGAATATAGATTTTTGGCGGTAGGTCACTAGTTTTGGTATTTCGTTATTATTTTTT  300
PI_209332    CACGTGAATATAGATTTTTGGCGGTAGGTCACTAGTTTTGGTATTTCGTTATTATTTTTT  300
Peking       CACGTGAATATAGATTTTTGGCGTTAGGTCACTAGTTTTGGTATTTCGTTATTATTTTTT  300

Wm82         TATTTCCAAGTAAATTGGATTAGAATATTTGAACTTCTTGGTTGCTGTCTCCTGGGTCAT  360
PI_507623    TATTTCCAAGTAAATTGGATTAGAATATTTGAACTTCTTTGTAGCTGTCTCCTGGGTCAT  360
PI_209332    TATTTCCAAGTAAATTGGATTAGAATATTTGAACTTCTTGGTTGCTGTCTCCTGGGTCAT  360
Peking       TATTTCCAAGTAAATTGGATTAGAATATTTGAACTTCTTTGTAGCTGTCTCCTGGGTCAT  360

Wm82         AATGTTTTATTATATTTTGGTATTAGCATAGCATTGTGATAGCACTATTACTATTTTGTT  420
PI_507623    AATGTTTTATTGTATTTTGGTATTAGCATAGCATTGTGATAGCACTATTACTATTTTGTT  420
PI_209332    AATGTTTTATTATATTTTGGTATTAGCATAGCATTGTGATAGCACTATTACTATTTTGTT  420
Peking       AATGTTTTATTGTATTTTGGTATTAGCATAGCATTGTGATAGCACTATTGCTATTTTGTT  420

Wm82         TGCTGATTCACTAGTACATTTGCCAGATGAAACTGACATTTTTTTTAATCCTGGTGGATA  480
PI_507623    TGCTGATTCACTAGTACATTTGCCAGATGAAACTGACATTTTTTTTAATCCTGGTGGATA  480
PI_209332    TGCTGATTCACTAGTACATTTGCCAGATGAAACTGACATTTTTTTTAATCCTGGTGGATA  480
Peking       TGCTGATTCACTAGTACATTTGCCAGATGAAACTGACATTTTTTTTAATCCTGGTGGATA  480

Wm82         GGACATTGCTGCTGCAATTGATGAAGAAGATGTTGCGAAGTTTACTGATGTTGTCAAGGA  540
PI_507623    GGACATTGCTGCTGCAATTGATGAAGAAGATGTTGCGAAGTTTACTGATGTTGTCAAGGA  540
PI_209332    GGACATTGCTGCTGCAATTGATGAAGAAGATGTTGCGAAGTTTACTGATGTTGTCAAGGA  540
Peking       GGACATTGCTGCTGCAATTGATGAAGAAGATGTTGCGAAGTTTACTGATGTTGTCAAGGA  540

Wm82         ATTTGATAGCATGACCCCTCTGGTAAGCTCCAAAAGTTGTTAAGTAGGATAACTTCTAGT  600
PI_507623    ATTTGATAGCATGACCCCTCTGGTAAGCTCCAAAAGTTGTTAAGTAGGATAACTTCTAGT  600
PI_209332    ATTTGATAGCATGACCCCTCTGGTAAGCTCCAAAAGTTGTTAAGTAGGATAACTTCTAGT  600
Peking       ATTTGATAGCATGACCCCTCTGGTAAGCTCCAAAAGTTGTTAAGTAGGATAACTTCTAGT  600

Wm82         GGTATTTAACAAAAATACTTCCACTGTATTTTTTATCCACATTTTATACAA  651
PI_507623    GGTATTTAACAAAAATACTTCCACTGTATTTTTTATCCACATTTTATACAA  651
PI_209332    GGTATTTAACAAAAATACTTCCACTGTATTTTTTATCCACATTTTATACAA  651
Peking       GGTATTTAACAAAAATACTTCCACTGTATTTTTTATCCACATTTTATACAA  651
```

Figure S5. Multicopy *rhg1-ds*-containing *Glycine soja* do not contain the chromosome 11 α-SNAP$_{Ch11}$ intron retention allele. Genomic sequence comparison of the α-SNAP$_{Ch11}$ region surrounding the previously described intron retention allele. Note that *G. max* PI 209332 carries *rhg1-b*. High copy (HC)-like single nucleotide polymorphisms highlighted in red and Peking-like SNPs highlighted in blue. The black box indicates the intron retention-associated SNP. Note that the sequence for *G. soja* PI 507623 was identical to the sequences for PCR-amplified genomic DNA obtained for the other six *rhg1-ds* accessions PI 342434, PI 378695A, PI 378695B, PI 407287, PI 507613 and PI 507614B. The SoySNP50K ss715610416 site associated with this intron-retention allele (Table S1; Bayless et al., 2018) is at a closely linked site not examined in the present experiment.

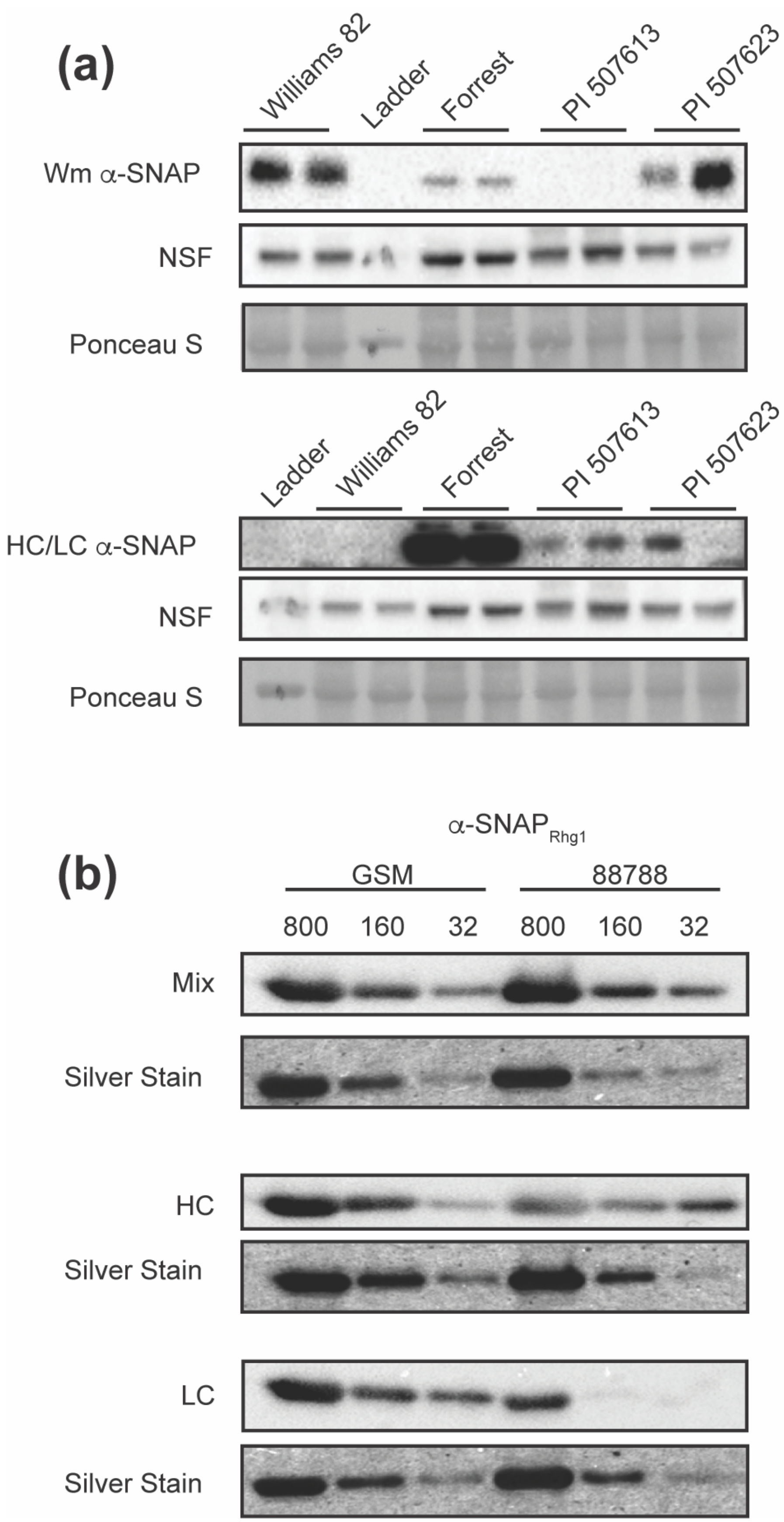
(a)
Williams 82
Ladder
Forrest
PI 507613
PI 507623
Wm α-SNAP
NSF
Ponceau S
Ladder
Williams 82
Forrest
PI 507613
PI 507623
HC/LC α-SNAP
NSF
Ponceau S
(b)
α-SNAP Rhg1
GSM
88788
800 160 32 800 160 32
Mix
Silver Stain
HC
Silver Stain
LC
Silver Stain

Figure S6. Some *rhg1-ds*-containing G*lycine soja* have lower apparent expression of multicopy-encoded α-SNAP$_{Rhg1}$Gsm and/or wild-type α-SNAP in developing trifoliates.
(a) Immunoblot of protein extracts from developing trifoliates, showing NSF, α-SNAP$_{WT}$ and α-SNAP$_{Rhg1}$ abundance in select *G. soja* varieties along with Williams 82 (Wm82) and Forrest controls. Polyclonal antibody used is listed to left of each set of lanes. Ponceau S shown as overall protein loading control. (b) Immunoblots showing relative detection of purified α-SNAP$_{Rhg1}$Gsm by custom antibodies, in comparison to detection of high copy α-SNAP (α-SNAP$_{Rhg1}$HC) by the same antibodies. Polyclonal antibody used is listed to left of each set of lanes; 'Mix' is anti-HC + anti-LC. Silver stain shown as loading control. The numbers 800, 160, 32 indicate pg of purified recombinant α-SNAP protein loaded in that lane.
